# Heritable viral symbionts in the family *Iflaviridae* are widespread among aphids

**DOI:** 10.1128/aem.01606-25

**Published:** 2025-10-30

**Authors:** Paula Rozo-Lopez, Vanesa Torres, Joseph Torres, Barbara S. Drolet, Simon Käfer, Benjamin J. Parker

**Affiliations:** 1 Department of Microbiology, University of Tennessee 189504, , Knoxville, Tennessee, USA; 2 Department of Biology, University of North Carolina at Chapel Hill 2331, Chapel Hill, North Carolina, USA; 3 Department of Entomology, Kansas State University 167081, Manhattan, Kansas, USA; 4 Institute of Biology and Environmental Sciences, Carl von Ossietzky Universität Oldenburg 11233, Oldenburg, Germany; Norwegian University of Life Sciences, Ås, Norway

**Keywords:** heritable viruses, *Iflavirus*, symbiosis, aphids

## Abstract

Heritable microbes shape host phenotypes and serve as important drivers of evolution. While interactions between insects and bacterial symbionts have been extensively studied, the prevalence and evolution of insect-viral symbioses remain poorly understood. We discovered multiple new species of iflaviruses in aphids, an important model for research on symbiosis, and found these microbes to be widespread across aphid species. We show that iflaviruses are persistently maintained in asexual host lines without apparent fitness costs while being transmitted vertically from mothers to offspring. Using field data and phylogenetic evidence, we found that aphid iflaviruses move horizontally among host species, but laboratory experiments showed that horizontal transmission does not result in persistent infections. Using quantitative PCR and immunohistochemistry, we discovered that viral infections localize in the host fat bodies and developing embryos. Surprisingly, we also found viral infections inside bacteria-housing cells called bacteriocytes, along with a positive correlation between viral and bacterial symbiont density. Together, our work suggests that iflaviruses are widespread heritable symbionts in aphids.

**IMPORTANCE:**

In recent years, the rise of metatranscriptome sequencing has led to the rapid discovery of novel viral sequences in insects. However, few studies have carefully investigated the dynamics of insect-virus interactions to produce a general understanding of viral symbiosis. Aphids are an important model for understanding the evolution and molecular basis of symbiosis, but whether viruses are forming persistent symbiotic relationships with aphids remains unclear. Here, we show that heritable iflaviruses are a widespread but previously unrecognized part of the aphid heritable microbiome. Aphid iflaviruses are transmitted alongside bacteria from mothers to offspring, potentially via specialized bacteriocytes that house symbiotic microbes. Our findings suggest that aphids establish persistent relationships with iflaviruses and are likely coevolving with these viral symbionts.

## INTRODUCTION

Our understanding of viruses associated with insects has historically focused on pathogens that infect economically important insects and viruses transmitted to humans, animals, or plants by insect vectors. However, the use of metatranscriptome sequencing over the last two decades has uncovered a vast diversity of novel viruses associated with insects (1–3). Some studies have also shown that specific viruses can persist across life stages and be inherited through multiple generations (4–6). The growing number of examples of these infections suggests that heritable viral symbiosis could be a widespread and integral aspect of insect biology (7), but research on these associations and their evolutionary significance remains limited.

In contrast, decades of studies have focused on interactions between insects and heritable bacterial symbionts that are primarily passed maternally from reproductive females to offspring (8). Due to the energetic costs that heritable bacteria impose on their hosts, they have evolved strategies for spreading through host populations either by conferring benefits or with reproductive manipulation (9, 10). Although there is some evidence of host-symbiont specificity, horizontal transmission of bacterial symbionts across insect taxa is a key component of infection dynamics in many systems (11, 12). To establish a similar ecological and evolutionary framework for insect-viral symbiosis, there is still a need to develop and study tractable model insect-virus systems.

Aphids (Hemiptera: Aphididae) are arguably one of the most important model insect systems for studying bacterial symbiosis (13). Nearly all 5,000 aphid species interact with *Buchnera aphidicola,* a bacterial symbiont that is required for host survival since it provides its hosts with amino acids missing from the diet. *Buchnera* and several other species of facultative bacterial symbionts are vertically transmitted from females to their offspring through asexual (parthenogenetic) and sexual phases of the aphid lifecycle (11, 14, 15). Heritable symbiosis has extensively shaped aphid evolution, with bacteria localized inside specialized cells that aphids evolved to house bacteria called bacteriocytes (16), which play a distinct role in vertical transmission (16).

Recent studies have identified species of viruses in the families *Bunyaviridae* (17), *Iflaviridae* (18), and *Parvoviridae* (19, 20) in laboratory stock lines of aphid hosts. The family *Iflaviridae* comprises a single genus (*Iflavirus*) of positive-sense single-stranded RNA (+ssRNA) viruses capable of infecting multiple insect orders such as Lepidoptera, Hymenoptera, Hemiptera, Diptera, Coleoptera, and Orthoptera. In some insects, iflaviruses are potent insect pathogens, while others exhibit asymptomatic persistent infections maintained by a combination of horizontal and vertical transmission (21). The genomes of three aphid iflaviruses have been sequenced: *Brevicoryne brassicae virus* (BrBV) (18), *Aphis aurantii iflavirus* (Iva) (22), and *Cavariella aegopodii 1* (CAIV1) (23), but little is known about their biology or transmission dynamics.

In this study, we used metatranscriptome sequencing of field-collected aphids and a bioinformatic screen of publicly available aphid transcriptomes to assemble genomes for multiple new strains and species of iflaviruses infecting hosts across the aphid phylogeny. We show that multiple species of iflaviruses persist in laboratory lines and are vertically transmitted from mothers to offspring. Using molecular screening, we show that specific viruses can be detected in multiple aphid species cohabiting on the same plant, but the infections are heritable only in specific hosts. Moreover, we demonstrate that the phylogenies of aphids and iflaviruses do not completely align, suggesting that cross-species horizontal transmission may occur on evolutionary timescales. We also found that these heritable infections have no clear negative fitness effects on aphids and are present in specific tissues like fat bodies, ovaries (developing embryos), and bacteriocytes. Lastly, we show that the density of viral infections correlates with bacterial symbiont titers, suggesting an interaction between these two microbes that could be key to future work. Together, our findings demonstrate that iflaviruses are widespread symbionts and suggest that heritable viruses may be hidden symbiotic partners that are coevolving with many insect taxa.

## RESULTS

### *Iflavirus* discovery using RNAseq

**Fig 1:**
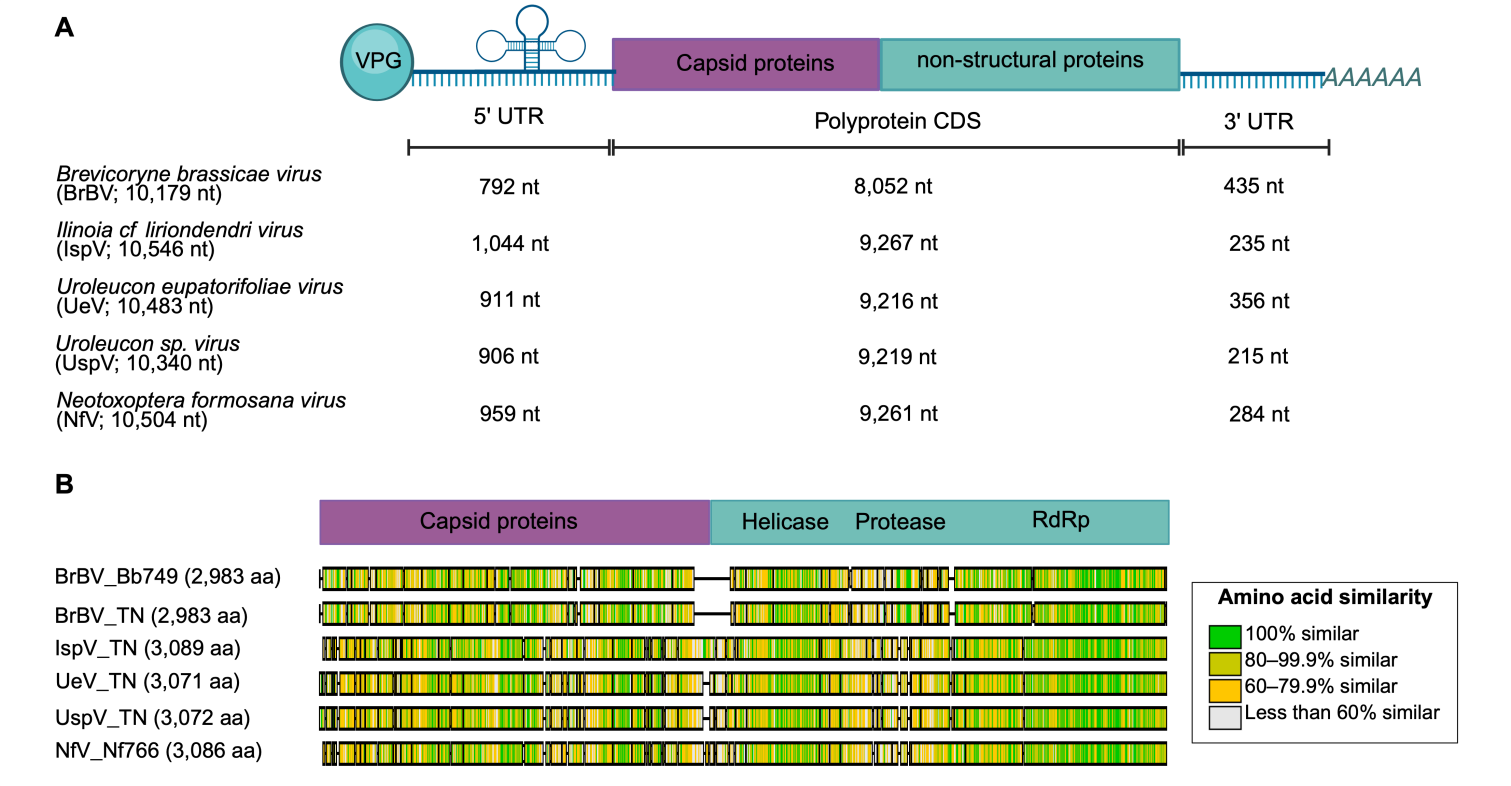
Newly identified aphid iflaviruses. (**A**) Complete consensus genome structure showing the nucleotide (nt) lengths of the polyprotein-coding region and the 5′ and 3′ untranslated regions (UTRs). (**B**) Alignment of polyprotein sequences displaying amino acid (aa) similarity with a color scale. Green indicates higher similarity per site, yellow indicates moderate similarity, and grey indicates low similarity. BrBV_TN, IspV_TT, UeV_TN, and UspV_TN are viral sequences obtained from metagenomic data of field-collected insects. BrBV_Bb749 and Nf_N766 are viral sequences detected in SRA data sets.

We discovered multiple new species of iflaviruses associated with aphid hosts through metatranscriptome sequencing of field-collected aphids. Initially, we established laboratory lines from field-collected parental aphids (P) of the tribe Macrosiphini (Wilson, 1910) and maintained them for at least three subsequent generations (F_1_–F_3_) (Table S1). Then, sequenced RNA from ribosomal RNA-depleted libraries for a subset of the parental lines (Table S2) and screened it for viral reads using a cloud-based open-source bioinformatics platform called CZ ID (Fig. S1; Table S2) and a consistency-based virus detection pipeline called TRAVIS. We selected viral read assemblies (>10,000 nt) that exhibited nucleotide and amino acid similarities to members of the family *Iflaviridae* (9–11 kb genomes encoding a single large polyprotein) and used the aphid *Brevicoryne brassicae virus* genome (BrBV; NCBI accession YP_001285409.1) as a reference to generate newly identified consensus genomes. Following the ICTV species demarcation criteria for iflaviruses, we classified genome assemblies obtained from different host species that showed a capsid protein (VP) amino acid sequence similarity below 90% as new viral species (24). Within the same virus species, genome assemblies from distinctive geographical locations are considered new viral strains. This approach produced complete genomes (99.9% coverage breadth) for a new strain of BrBV (genome size of 10,179 nt) and three new *Iflavirus* species, named *Ilinoia iflavirus* (IspV; 10,546 nt)*, Uroleucon eupatarifoliae virus* (UeV; 10,483 nt), and *Uroleucon iflavirus* (UspV; 10,340 nt) (Fig. 1A; Table S3).

### *Iflavirus* discovery in the Sequence Read Archive

In addition to our direct sequencing efforts, we identified further evidence of widespread *Iflavirus* prevalence in existing data sets within the Sequence Read Archive (SRA). After screening 27 RNAseq data sets from 22 species (Table S4), we assembled a sequence for a new *Iflavirus* species named *Neotoxoptera formosana virus* (NfV; 10,504 nt), and an additional strain of BrBV (UK strain; genome size of 10,179 nt) (Table S3). We also obtained a 7,092 nt sequence of the coding region from a potentially new *Iflavirus* hosted by *Megoura crassicauda* (named *Megoura crassicauda virus*; McV), but it is not included in further analysis due to the absence of a complete polyprotein sequence.

All six newly assembled *Iflavirus* genomes have a single large ORF (CDS 8,952–9,267) flanked by a 5′ untranslated region (5′ UTR), which includes an internal ribosome entry site (IRES, required for the cap-independent translation) and a 3′ UTR with a poly(A) tail that terminates translation (21) (Fig. 1A). The amino acid similarity of the newly assembled aphid iflaviruses polyproteins is the highest at the RNA-dependent RNA polymerase (RdRp) region (Fig. 1B), while the overall polyprotein similarity ranged from 33.32% to 87.96% (Table S5).

### Phylogenetic analysis of aphid iflaviruses

**Fig 2:**
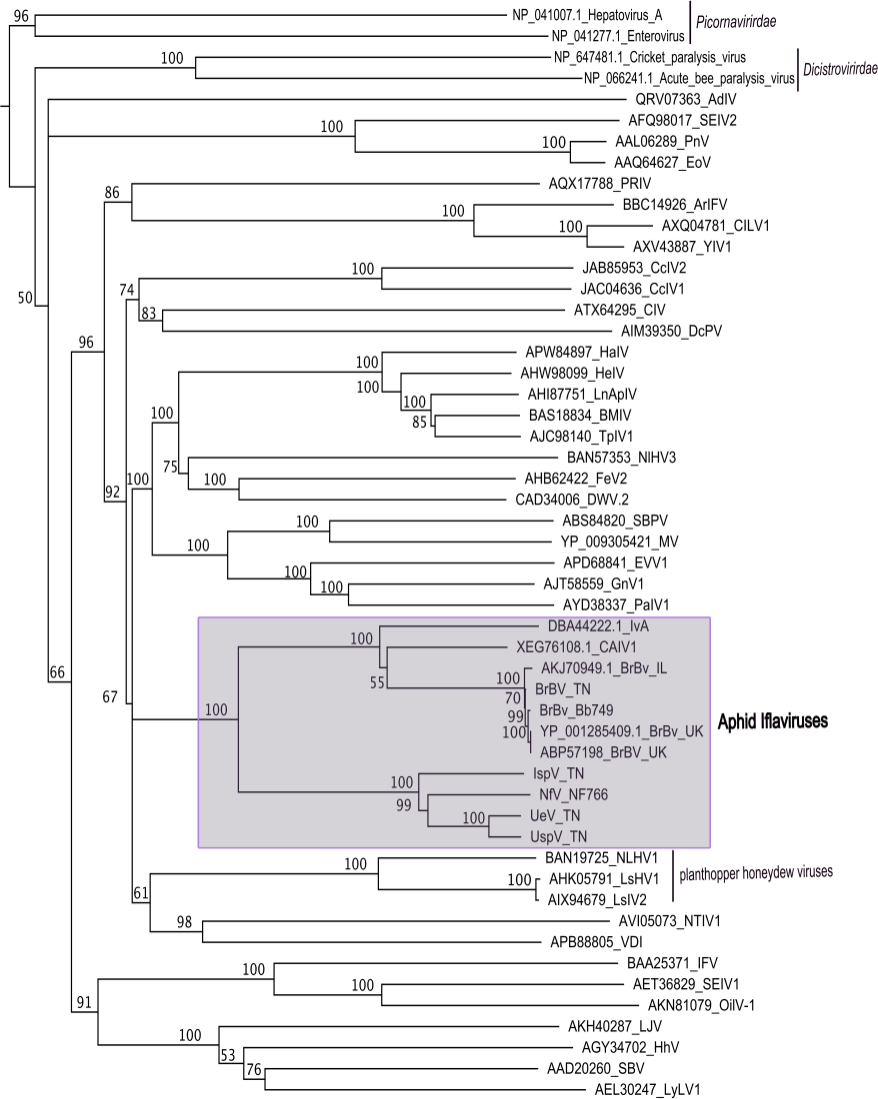
Evolutionary analysis of aphid iflaviruses and ICTV-recognized members of *Iflaviridae*. The phylogenetic tree was constructed using the Maximum-likelihood method with the protein gamma JTT matrix model based on an MAFFT alignment (FFT-NS-i_x1000, BLOSUM62, and 1.54 gap penalty). Families *Dicistroviridae* and *Picornaviridae* are used as outgroups. Bootstrap values on each node indicate the percentage from 500 replicates.

We found that aphid iflaviruses form a monophylogenetic clade related to other Hemipteran iflaviruses. We used a Maximum-likelihood tree based on an alignment of the polyprotein sequences of all available aphid iflaviruses and 37 reference species (Fig. 2). Within the aphid iflaviruses clade, there are two distinctive groups with 100% bootstrap support. Group 1 contains all BrBV strains, *Aphis aurantii iflavirus* (Iva), and *Cavariella aegopodii 1* (CAIV1), whereas group 2 includes *Uroleucon* iflaviruses (UeV and UspV), IspV, and NfV. We are confident of the monophyletic nature of aphid iflaviruses, as well as their clustering patterns, because they were reproducible by other methods such as neighbor-joining (Fig. S2) and even when we included an additional 237 curated sequences from a wide range of iflaviruses infecting arthropods (Fig. S3). Likewise, across all phylogenies, we observed a clade of planthopper (*Nilaparvata lugens* and *Laodelphax striatellus*) honeydew viruses as the closest related group to the aphid iflaviruses clade.

### *Iflavirus* prevalence in field samples

We found that individual *Iflavirus* species can be detected in multiple host aphid species and are found at intermediate frequencies. We tested the prevalence of iflaviruses, identified in RNAseq data of field-collected aphids, across the parental (P) aphid lines using PCR with primer sets that we designed and validated to be specific to each viral species (Table 1). We detected BrBV in both field-collected *B. brassicae* (72.2%) and *Lipaphis pseudobrassicae* (16.7%) from *Brassica* plants. Similarly, we found UeV with 100% prevalence in *Uroleucon eupatoricolens* and in a lower percentage (20%) of different morphotypes of aphids from the genus *Uroleucon* (large daisy aphids), whose species assignment could not be confirmed due to the lack of progeny used to obtain functional barcode sequences. Likewise, UspV infections ranged from 0% to 100% across multiple species of aphids in the genus *Uroleucon* collected on Asteraceae. We also detected iflaviruses infecting a single aphid host species, as in the case of IspV found in 22.2% of *Ilinoia liriodendri* aphids from tulip trees (*Liriodendron tulipifera*).

**TABLE 1:** Aphid iflavirus prevalence in field aphids and heritability in laboratory lines

Iflavirus	Aphid species	Percentage of *Iflavirus*-positive lines	
		Parental (P)	Progeny (F_1_–F_3_)
BrBV	*Brevicoryne brassicae* (Bb)	72.2% (13 of 18)	100% (11 of 11)
	*Lipaphis pseudobrassicae* (Lp)	16.7% (1 of 6)	0% (0 of 1)
IspV	*Ilinoia liriodendri* (Il)	22.2% (4 of 18)	100% (3 of 3)
UeV	*Uroleucon eupatoricolens*	100% (3 of 3)	100% (2 of 2)
	*Uroleucon* spp.	20% (1 of 5)	NC^*a*^
UspV	*Uroleucon eupatoricolens*	33.3% (1 of 3)	NC
	*Uroleucon nigrotuberculatum*	0% (0 of 1)	NC
	*Uroleucon rurale*	100% (2 of 2)	100% (2 of 2)
	*Uroleucon* spp.	100% (5 of 5)	NC

^*a*^ NC: not colonized; aphids died before reaching three generations in the laboratory.

### *Iflavirus* persistence in laboratory lines

We showed that *Iflavirus* infections are maintained in laboratory lines and are vertically transmitted from females to offspring across at least three generations, a key benchmark for determining heritability (25). We assessed *Iflavirus* heritability in laboratory lines of the virus-positive parental strains by PCR testing individual adults one (F_1_) and three (F_3_) generations after laboratory colonization (Table 1). All four *Iflavirus* species were maintained with 100% fidelity. Unlike *Uroleucon*, *B. brassicae* and *I. liriodendri* lines are easily maintained in the laboratory, with BrBV and IspV further showing stable heritable infections for more than 30 generations (over 1 year). Therefore, we used these aphid-virus pairings for subsequent transmission experiments.

### *Iflavirus* vertical transmission in laboratory lines

We found evidence of vertical transmission of BrBV and IspV from mothers to offspring. *B. brassicae* and *I. liriodendri* females vertically transmit their respective iflaviruses to their offspring, but the proportion of infected offspring (collected immediately as they were born from a single *Iflavirus*-positive female) varied across species and lines. We detected iflaviruses by PCR in 84.6% (±0.11) of *I. liriodendri* progeny and 98.7% (±0.2) to 43.7% (±0.11) of *B. brassicae offspring*, depending on the line. We did not find significant differences (Mann-Whitney test, *P* = 0.9591) in relative BrBV adult body titers (qPCR) that could explain the difference in the percentage of virus-positive progeny between *B. brassicae* lines (Fig. S4). To produce a parthenogenic BrBV(–) line, we split the line with the lowest number of virus-positive progeny into multiple sublines and maintained them for five generations at lower aphid densities while randomly testing 1–3 adults at each generation. Although we observed some PCR-negative adults, our qPCR analysis revealed that those lines still retained the virus at titers below the conventional PCR limit of detection, and BrBV was detectable again by PCR when aphids were subjected to crowded conditions.

### Within species horizontal transmission of iflaviruses

**Fig 3:**
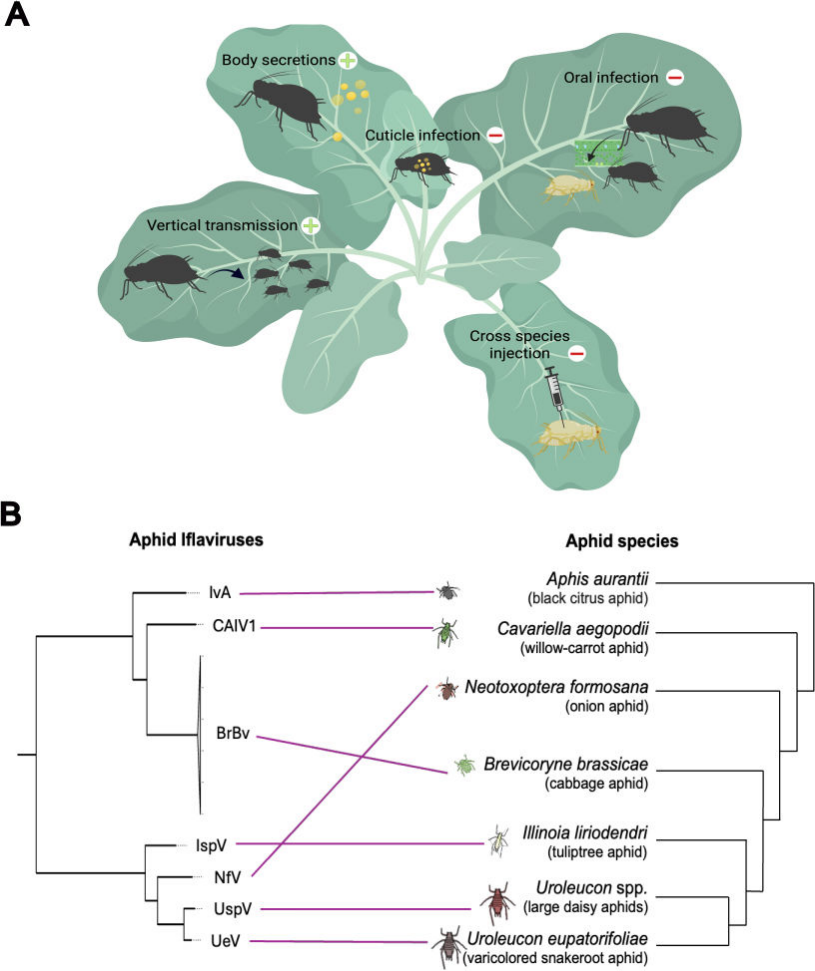
Transmission and host association of aphid iflaviruses. (**A**) Experiments show that vertical transmission of aphid iflaviruses is species-specific and that horizontal exposure does not produce laboratory heritable infections within lines of the same species or across distantly related aphids. (**B**) Representation of phylogenetic trees of aphid iflaviruses (left) and aphid species (right; based on reference 26), with purple lines indicating which hosts harbor sustained infections of a particular Iflavirus.

Through a series of experiments, we found that vertical transmission of aphid iflaviruses is species-specific and that horizontal exposure does not produce heritable infections among laboratory lines of the same host species (Fig. 3A). Unlike the variable proportion of infected offspring detected as they were born, we observed that 100% of F_1_ adults were PCR-positive for BrBV or IspV infections when allowed to cohabitate with *Iflavirus*-positive reproductive females for 4 days on leaf cuttings before being transferred to new host plants. We also found that honeydew and cornicle droplets collected from *B. brassicae* and *I. liriodendri* adult aphids and PBS washes of plant surfaces contaminated with body secretions were qPCR-positive for BrBV and IspV, respectively (Fig. S5), suggesting that transmission of iflaviruses during cohabitation may be enhanced by virus-positive body secretions.

To investigate whether intraspecific horizontal transmission contributes to the maintenance of aphid iflaviruses, we exposed *Iflavirus*-negative *I. liriodendri* aphids to IspV-positive aphid homogenates both topically and orally. We detected viral transmission through the insect cuticle in 10% (qPCR; 1 out of 10 pools) of the adults that were topically exposed as nymphs to IspV(+) homogenates. However, none of their progeny tested positive for IspV infections (*n* = 12 pools), indicating that while Iflavirus spread through the cuticle may occur at low rates, it does not lead to heritable infections. Additionally, we found that 100% (*n* = 5 pools) of nymphs were PCR-positive for IspV immediately after feeding for 24 h on artificial diets supplemented with IspV(+) homogenates. Yet, after transferring the aphids to virus-free plants to remove ingested virus that was not acquired, none tested positive for IspV infections 2 (*n* = 5 pools) or 7 days (*n* = 3 pools) later. This suggests that the ingested virus does not produce active infections. Similarly, 4.8% of nymphs (1 out of 21) were PCR-positive for IspV after feeding for 3 days on IspV(+) homogenates delivered through the host plant roots. As before, none of the IspV-fed aphids that reached adulthood on virus-free plants (*n* = 18), nor their progeny (*n* = 18 pools), tested positive for IspV infections, indicating that *Iflavirus* spread via the plant phloem does not result in heritable infections.

### Cross-species transmission of iflaviruses

We also found that horizontal exposure does not produce heritable infections in distantly related aphids (Fig. 3A). Driven by our field data, we explored whether BrBV is horizontally transmitted from *B. brassicae* [BrBV(+)] to *L. pseudobrassicae* [BrBV(–)] infesting the same host plants. Initially, we found that 75% (9 out of 12) of *L. pseudobrassicae* were PCR-positive after cohabitation with BrBV(+) aphids on leaf cuttings. After transferring the BrBV(+) adults to virus-free plants in the absence of virus-positive aphids, we observed that all the offspring produced were qPCR-negative for BrBV infections (*n* = 31), suggesting that *L. pseudobrassicae* reproductive females may have tested positive for BrBV after cohabitation because of surface contamination with *B. brassicae* body secretions rather than active heritable infections.

When we tested the potential for oral cross-species transmission of BrBV through the host plant, we found that 50% (2 out of 4) of *L. pseudobrassicae* were PCR-positive for BrBV after the initial feeding on the virus-plant interface, but none were virus-positive after reaching adulthood on virus-free plants (qPCR; *n* = 29). Overall, our data suggest that BrBV oral infections do not result in persistent infections in *L. pseudobrassicae*. We also injected BrBV directly into *L. pseudobrassicae* aphids to bypass all infection barriers and found that 100% of aphids (*n* = 3 nymphs) tested positive for BrBV by PCR immediately after injection, and 27% (3 out of 11 adults) remained positive three days post-injection. However, we did not detect BrBV in injected *L. pseudobrassicae* after seven days post-injection (*n* = 7 adults), nor in their progeny (*n* = 6 pools of nymphs). These findings suggest that BrBV has a specific host range, and the *L. pseudobrassicae* parental aphids testing positive for BrBV in our field survey likely represent cross-contamination of viral RNA rather than active infections.

Lastly, we compared our phylogeny of aphid iflaviruses with the most recent molecular phylogeny of Macrosiphini aphids (26) and found that the virus evolutionary relationships recapitulate some, but not all of the clustering patterns of aphid hosts (Fig. 3B). These phylogenetic incongruences indicate that host switching and/or horizontal transmission events across host species may occur on evolutionary time scales.

### *Iflavirus* influence on aphid fitness

We observed that iflaviruses are maintained in laboratory lines without apparent fitness costs to aphids. We used IspV(+) and IspV(−) *I. liriodenri* lines to evaluate the effects of *Iflavirus* infection on aphid life history traits although it is important to note that these are not genetically identical aphid lines. We found no significant differences in fecundity (Mann-Whitney test, *P* = 0.4502; Fig. S6A), with a single IspV(+) parental female producing an average of 9.33 (±4.13) offspring and an IspV(–) female producing 7.5 (±2.42). Neither the developmental time (12.5 ± 0.84 days for both lines; *P* > 0.999; Fig. S6B) nor stress-induced wing production after starvation and crowding (0% winged offspring for both lines; *P* > 0.9999; Fig. S6C) was affected by virus status. Our results suggest that symbiotic *Iflavirus* infections do not influence these fitness traits of aphids maintained in the laboratory.

### *Iflavirus* tissue tropism

**Fig 4:**
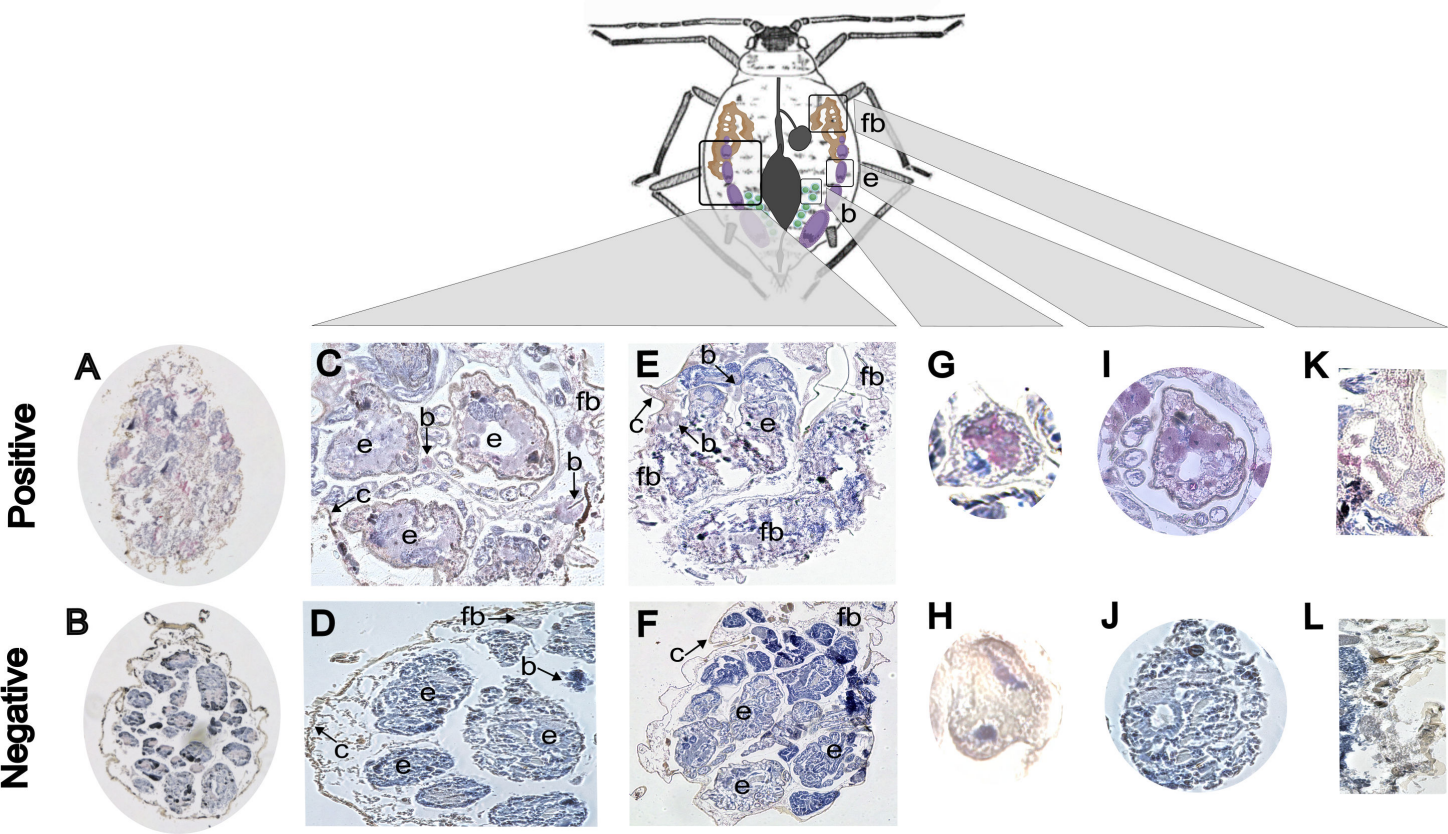
Immunohistochemistry staining of BrBV-infected aphid tissues. (**A**) Hematoxylin-stained longitudinal sections of the whole *B. brassicae* and (**B**) *L. pseudobrassicae*. (**A, C, D, E, G, I, K**) Longitudinal sections of *B. brassicae* tissues showing BrBV-positive (pink) staining. (**B, D, F, H, J, L**) Longitudinal sections of *L. pseudobrassicae* tissues were used as negative-staining controls. (**G**) BrBV-positive and (**H**) BrBV-negative bacteriocyte. (**I**) BrBV-positive and (**J**) BrBV-negative embryo. (**K**) BrBV-positive and (**L**) BrBV-negative fat body. All sections were counterstained with hematoxylin (blue) to reveal the overall tissue structure. Abbreviations: b: bacteriocyte, c: cuticle, e: developing embryo, fb: fat body.

We found that BrBV is distributed across multiple aphid tissues and with infection patterns relevant to vertical transmission. We detected BrBV via qPCR in dissected *B. brassicae* heads, digestive tracts (with bacteriocytes attached), developing embryos, and carcasses (with fat body attached), with carcasses showing the highest relative viral titers, although not statistically significant (Kruskal-Wallis test, *P* = 0.0729; Fig. S7). It is important to note that material for the tissue examination was pooled from several individuals and could be cross-contaminated to some degree by body secretions released during the dissection process. For a more precise description of *Iflavirus* tissue tropism, we used immunohistochemistry to visualize the BrBV infection patterns (pink staining) on longitudinal sections of adult *B. brassicae* (Fig. 4A, C, D, E, G, I and K) in comparison with BrBV-negative *L. pseudobrassicae* sections (Fig. 4B, D, F, H, J and L). We observed positive intracellular staining in 81.8% of the examined aphids (18/22), with fat bodies being the most prevalent positive tissue (positive signal detected in 72.7% of aphids examined) (Fig. 4C, E and K), followed by developing embryos (63.6%) (Fig. 4C, E and I), and bacteriocytes (22.7%) (Fig. 4C and G). No positive staining was observed in the brain, hindgut, or midgut, suggesting these tissues may be refractory to BrBV infection.

### Correlation with *Buchnera* density

**Fig 5:**
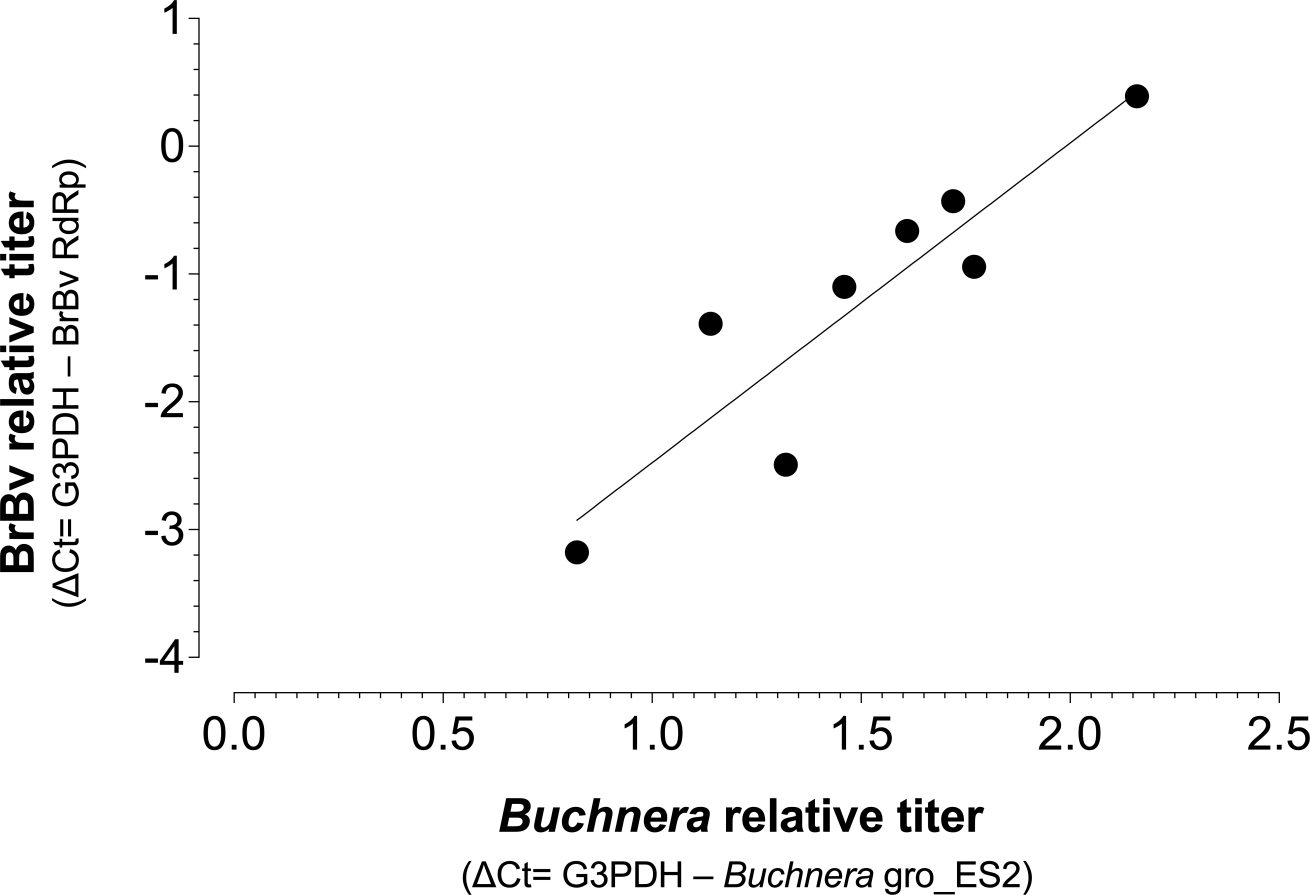
Correlation between viral (BrBV) and bacterial (*Buchnera*) symbionts relative titers (qPCR). Spearman rank-order correlation, *r* = 0.9048, *P* = 0.0023. The aphid reference gene G3PDH was used to normalize the data.

Based on positive *Iflavirus* staining inside bacteriocytes described above, we measured the within-host density of the obligate bacterial symbiont *Buchnera aphidicola* across *B. brassicae* aphids with different BrBV titers. We found a positive correlation between the relative titers of bacterial and viral symbionts (Fig. 5; Spearman Rank-Order correlation, *r* = 0.9048, *P* = 0.0023). One possible explanation is that this trend is driven by the colocalization of both microbes in bacteriocytes, with bacteriocyte numbers potentially varying across generations (27).

## DISCUSSION

We integrated metatranscriptomic and molecular screening with experimental assays to investigate *Iflavirus* infections across aphid species. Our results revealed that iflaviruses have a long history of interaction with the aphid clade and are an unrecognized component of these insects’ heritable microbiome, where the symbiosis is shaped by strong vertical transmission, but viruses likely move across host lineages over evolutionary timescales.

Aphid iflaviruses appear as asymptomatic, species-specific, heritable infections with fluctuating titers. Phylogenetically, the aphid-*Iflavirus* association may have been ancestral to the group related to honeydew viruses infecting other hemipterans, suggesting that body secretions could facilitate low-rate horizontal transmission, as indicated by the phylogenetic incongruence of *Brevicoryne* and *Neotoxoptera* iflaviruses. In some hemipterans, infectious honeydew penetrates the cuticle of immature insects and passes through the tracheae to replicate within the insect body (28). However, our results show that infrequent contact transmission of *I. liriodendri* does not lead to heritable infections of IspV. Similarly, honeydew transmission of BrBV does not establish active infections in distantly related aphids (*L. pseudobrassicae*) sharing Brassica plants with virus-positive hosts (*B. brassicae*). Further research is needed to determine whether the *Uroleucon* iflaviruses found in our field survey can replicate across multiple closely related hosts or if they are in early stages of diversification, with different viral strains infecting specific host species.

Other aphid heritable viruses (*Parvoviridae: Densovirus*) have been shown to systemically spread through the plant phloem after saliva injection (20, 29). Although BrBV replication does not occur in the plant leaves hosting infected aphids (18), we detected relatively high viral loads on plant surfaces and found that iflaviruses can travel through plant tissues. *Iflavirus* oral ingestion by aphids occurs within 1–3 days, but virus-positive insects typically exhibit low viral titers that quickly fade after a gut-clearing period. Since ingested virions do not produce active heritable infections, and we did not observe positive staining in the digestive tract, it seems unlikely that iflaviruses can infect midgut epithelial cells.

The high heritability rates observed in our experimental assays could be favored by laboratory-rearing conditions (e.g., shared housing environments) that may increase virus titers and transmission opportunities through postoviposition secretions (18, 30, 31). However, we carried out exhaustive experimental assays that explored different routes for horizontal transmission to demonstrate that aphid iflaviruses are true heritable symbionts. Additionally, our examination of tissue infection patterns inside developing embryos and bacteriocytes confirmed that aphid iflaviruses are vertically transmitted from mothers to offspring. Although tissue infection varies among insect-virus pairings, ovarian tissue infection appears to be a common feature among iflaviruses infecting Hemiptera (28, 32). While further research is necessary to determine the transfer mechanisms by which iflaviruses are incorporated into developing embryos, positive viral staining inside the bacteriocytes suggests that aphid iflaviruses could co-opt essential maternal transmission mechanisms used by obligate and some facultative bacterial symbionts (33). In aphids, *Buchnera aphidicola* cells are transferred from bacteriocytes (which cluster near the germ cells) directly to the oocyte via cell-to-cell transfer from follicle cells during sexual reproduction and through exo/endocytosis during parthenogenetic embryogenesis (34). Alternatively, viral particles might bind to the outer membrane of bacterial symbionts to exploit their oocyte entry pathway, as seen in another system (35). We also localized iflaviruses in the fat bodies, which serve as important endocrine tissues crucial for biosynthetic and metabolic activity. Fat bodies provide a suitable niche for the proliferation of endosymbionts (36–41), with some infections altering their host’s glucose and glycogen metabolism through insulin signaling (42). Additionally, the fat body plays a vital immune role during viral infection (43), and the high load of BrBV (fat body positive staining and carcass qPCR titers) detected might help maintain immune homeostasis necessary for persistent infections (44, 45).

Lastly, we found no apparent detrimental effects of symbiont infection on the aphids’ fecundity, developmental times, or stress-induced wing production, but future studies are needed to evaluate potential context-dependent effects (e.g., environmental and biological stressors). More importantly, a controlled analysis of infections in genetically identical lines—when curing or artificial infection becomes possible—would allow us to identify the fitness consequences of viral infections. In viruses with broader host ranges, such as in the case of potential cross-species transmission of *Uroleucon* viruses, increased virulence due to maladaptation in the new host might suggest that asymptomatic infections only occur in the original host species (46).

Overall, our study provides fundamental insights into the evolutionary dynamics of heritable insect-virus interactions, specifically highlighting the persistent coevolutionary relationships between iflaviruses and aphid species. While metatranscriptome sequencing has led to the discovery of numerous viruses, the understanding of whether these viruses form long-term associations with their insect hosts has been limited. Our findings suggest that iflaviruses are not merely transient infections but are integrated into the biology of aphids, forming persistent infections across multiple species. Iflaviruses are transmitted vertically along with bacterial symbionts, indicating a deep species-specific interaction with their hosts. These findings suggest that iflaviruses should be studied in the same context as bacterial symbioses, which have been extensively researched in aphids. Our perspective opens new avenues for understanding the role of viruses in insect symbiosis and co-evolution.

## MATERIALS AND METHODS

### Aphid collection and colonization

We collected adult winged and wingless asexual female aphids infesting different host plant species across urban and agricultural landscapes in East Tennessee, USA, between April–June and October–December 2022–2024 (Table S1). We placed single aphids from the field in Petri dishes containing a leaf disc of the appropriate host plant and allowed them to reach parthenogenic reproduction in the laboratory. Then, collected surviving parental adults (P) that had produced at least five offspring and stored each parental aphid individually in Eppendorf tubes at −80°C until further processing. We transferred generation one (F_1_) nymphs (offspring produced in the Petri dishes) onto laboratory-grown, virus-free host plants and reared them under standard conditions (22°C, 16L:8D, and 45% relative humidity). Every 2 weeks, we transferred a new generation of nymphs onto fresh laboratory-grown plants (see Table S6 for laboratory host plant details), collected 1–3 adults per generation in Eppendorf tubes, and stored them at −80°C. We identified each aphid species as previously reported (47, 48) by DNA extraction, PCR amplification, and Sanger sequencing of the COI barcode region. Barcoded sequences are available in NCBI with accession numbers PV016919–PV016931.

### Total RNA extraction

We macerated −80°C-frozen aphids, tissues, body secretions, or homogenates with a pestle in 300 µL of TRIzol (Invitrogen; Thermo Fisher Scientific, Inc., Waltham, MA, USA) with 100 µL BCP (1-bromo-3-chloropropane; Life Technologies, Thermo Fisher Scientific, Inc., Waltham, MA, USA) and isopropanol precipitation. We resuspended each RNA pellet in 40 µL nuclease-free water, removed the genomic DNA with Zymo DNase I Reaction Mix according to the manufacturer instructions (Zymo Genetics Inc., Seattle, WA, USA), and cleaned and concentrated with the Zymo RNA Clean & Concentrator kit (Zymo Genetics Inc.) under recommended conditions.

### Metatranscriptome sequencing

We pooled equal amounts of RNA from between 1 and 6 aphids (P) of the same species (Table S2) to perform metatranscriptome sequencing at Novogene (Novogene Corporation Inc., Sacramento, CA, USA). Library preparation was conducted using ribosomal RNA (rRNA) depletion by Illumina TruSeq Stranded Total RNA with Ribo-Zero Plus and NEBNext rRNA Depletion Kit. Libraries were sequenced to approximately 9 billion base pairs (bp) per sample with 150 bp paired-end reads on an Illumina NovaSeq platform. Raw reads were deposited into the NCBI Sequence Read Archive under BioProject ID PRJNA1216975, accession numbers SRX27542651–SRX27542654.

### *Iflavirus* detection in RNAseq data

Using the CZ ID platform (https://czid.org) with Illumina mNGS Pipeline v8.2 (49, 50), we removed aphid-specific reads using the *Acyrthosiphon pisum* genome (51) and added the CZ ID water background model after processing. We identified the potential viral reads matching iflaviruses and unclassified RNA viruses using *z*-score metrics (≥1), alignment length over 50 matching nucleotides (NT L ≥ 50), and a minimum of five reads per million aligning to the reference protein database (NR rPM ≥ 5) (49, 52, 53). We confirmed the taxonomic classification of viral contigs with manual searches using BLASTX and BLASTN (NCBI). In addition, we downloaded non-assembled reads matching *Iflaviridae* or unclassified RNA viruses (as a proxy to capture putative Picorna-like viruses with potential *Iflaviridae* polyprotein motifs) and obtained additional viral contigs using Geneious *de novo* assembler at medium sensitivity (Geneious Prime v.2023.2.1).

We conducted an additional screening that searched for *Iflavirus* open reading frames (ORFs) using TRAVIS (v.20221029, https://github.com/kaefers/travis) on *de novo* transcriptome assemblies generated on Trinity v.2.15.1 using default settings (54). Our *Iflaviridae* reference database included the accepted viral species by the International Committee on Taxonomy of Viruses (ICTV) by 24 November 2023 (Table S7). Potential *Iflavirus* ORFs (100–3,000 amino acids) were extracted from the assembled transcriptomes and screened using HMMER v3.3.1 (55), MMSeqs2 (56), BLASTP v2.12.0 (57), and Diamond v2.0.15 (58) with an *e*-value cutoff of 1 × 10^−6^. All hits were again searched with Diamond against the NCBI non-redundant protein database.

### *Iflavirus* genome analysis, coding region, and polyprotein sequence

We used the CZ ID viral consensus genome pipeline to build a consensus genome for *Brevicoryne brassicae virus* (BrBV) by aligning the *Iflavirus* reads to the reference BrBV genome (NCBI accession NC_009530.1). For the other *Iflavirus* genomes, we aligned CZ ID and *de novo* assembled *Iflavirus* and Picorna-like contigs (>1,000 nt) with the viral contigs provided by TRAVIS using the Geneious Prime Clustal Omega v.1.2.3 plugin and mBed algorithm. We chose alignments with pairwise identity (over 99.7%) and nucleotide length over 9,000 as consensus sequences. NCBI ORF finder with standard genetic code (https://www.ncbi.nlm.nih.gov/orffinder) was used to identify the coding sequence (CDS) and the polyprotein translation. *Iflavirus* consensus genomes are available in NCBI (BrBV_TN accession PV031990, IspV_TN PV023937, UeV_TN PV023938, UspV_TN PV023939).

### *Iflavirus* detection in SRA data

We searched available RNAseq data sets from aphid species in the Sequence Read Archive (SRA) available through NCBI (3 March 2024). We selected non-pea aphid experiments from field-collected aphids sequenced with the Illumina platform as paired-end reads and with mixed selection methods and chose one experiment per species (Table S4). The accession numbers (SRX) were used to obtain their corresponding run number (SRR) into a custom bash pipeline to download corresponding read files using the SRA Toolkit (https://github.com/ncbi/sra-tools/wiki). Read files submitted to NCBI as multiple interleaved files were downloaded as individual read files and concatenated into two separate files (R1 and R2) with a cat command line. We conducted CZ ID and TRAVIS analyses as above and used NCBI ORF finder to identify the CDS and the polyprotein translation. Host species taxonomy (>99.98% identity) from which we obtained viral sequences was confirmed by blasting reference COI barcode sequences (http://www.boldsystems.org/) against the SRA experiment set. The *Iflavirus* consensus genomes and polyprotein sequences obtained from SRA have been deposited in NCBI (BrBV_bb749 accession BK070018, NfV_Nf7766 BK070016, McV BK070017).

### Phylogenetic analysis of iflaviruses

We aligned the polyprotein sequences of the newly and previously reported aphid iflaviruses (Table S8) and 37 reference *Iflavirus* species recognized by the ICTV (Table S9) in MAFFT v7.490 using FFT-NS-i_x1000 algorithm, matrix BLOSUM62, and 1.54 gap penalty (59). Then, used the polyprotein alignment to generate a Maximum–Likelihood tree using RaxML v8.2.12 with PROTGAMMAJTTF model and a majority rule bootstrap with 500 reps (60). To evaluate the consistency of clustering patterns among aphid iflaviruses, we also used polyprotein alignments (produced as above) to generate a Neighbor-Joining tree using the Jukes-Cantor distance model and 500 bootstrap replicates in Geneious Prime v2024.0 and an extended Maximum–likelihood tree that included 221 additional curated *Iflavirus* sequences (Table S10). Polyprotein sequences from *Dicistroviridae* (*Acute bee paralysis virus* NP_066241.1, and *Cricket paralysis virus* NP_647481.1) and *Picornaviridae* (*Enterovirus* C NP_041277.1NP_041277.1, and *Hepatovirus* A NP_041007.1) served as outgroups in all analyses.

### *Iflavirus* screening via PCR

We generated specific primer pairs targeting different regions encoding for the *Iflavirus* polyprotein of BrBV, IspV, UeV, and UspV (Table 2). Primer target specificities were confirmed by Sanger sequencing amplicons and aligning them to each *Iflavirus* genome. We also confirmed the species specificity of each primer set by retesting all virus-positive parental samples (based on RNAseq analysis) against the entire virus panel (six primer sets). For *Iflavirus* screening, 800 µg of total RNA (extracted as above) was used for cDNA synthesis using iScript cDNA synthesis kit (Bio-Rad Laboratories, Inc., Hercules, CA, USA), which uses random hexamer primers. Then, 1 µL undiluted cDNA was used as a template in a 25 µL PCR using Quick-Load Taq 2X Master Mix (New England Biolabs, Ipswich, MA, USA), 0.2 µM of each primer, and the following the thermal cycling conditions: 94°C for 5 min followed by 35 cycles of 94°C for 30 s, the corresponding annealing temperature for 30 s, 72°C for 30 s, and a final extension at 72°C for 3 min. We visualized PCR products in 1% agarose gels stained with SYBR Safe (Invitrogen, Inc.).

**TABLE 2:** Primers used for aphid *Iflavirus* screening using conventional PCR

Primer	Sequence (5′ to 3′)	Target	Amplicon (bp)	Annealing temp.
BrBV_Hel_F	ATCGCCGTCGTCATCAACTT	*Brevicoryne brassicae Iflavirus* (Helicase region)	500	66.5°C
BrBV_Hel_R	AACGGCGGCATTCTCTTCAA			
BrBV_RdRp_F	GGTACAGAACCCGGTCCTCT	*Brevicoryne brassicae Iflavirus* (RdRp region)	500	66.5°C
BrBV_RdRp_R	AAAACGCGCACTTTACCAGG			
IspV_VP_F	ACGACGCACCGGAGTATTAC	*Illinoia liriodendri Iflavirus* (VP region)	500	58°C
IspV_VP_R	ATGGGGCAACTGGTACCTTC			
IspV_RdRp_F	GAGGATATCGCCGAACGCTT	*Illinoia liriodendri Iflavirus* (RdRp region)	500	63°C
IsPV_RdRp_R	GCGGTAGGAAGGAGGAAACC			
UeV_2R	CAGCATATCGGTGAGTCCCC	*Uroleucon eupatoricolens virus* (RdRp region)	720	53°C
UeV_2F	TTCGGACCATGCTTTAGCGT			
UspV_F	ACAGGCGAGCAAATCTGGAA	*Uroleucon* sp. *virus* (Helicase region)	460	62°C
UspV_R	TGATGCCGTTTTTGGTGCAG			

### *Iflavirus* titer via qPCR

We used quantitative PCR (qPCR) to calculate relative viral titers with specific primers for BrBV and IspV (Table 3) and the aphid Glyceraldehyde 3-phosphate dehydrogenase (G3PDH) as an endogenous control gene for normalization of quantitative data and as an internal control of cDNA quality (61). We used 1 µL of undiluted cDNA (synthesized as above) as a template in a 20 µL reaction containing 1× PCR Buffer, Mg^2+^ (2 µM), dNTP mix (0.2 µM), and Taq (0.025 units/μL) (Invitrogen, Inc.) with 1× EvaGreen Dye (Biotium, Inc., Fremont, CA, USA) and the corresponding primer concentrations (Table 3; see Fig. S8 for efficiency). The qPCR parameters were an initial step of 95°C for 3 min and 40 cycles of 95°C for 10 s, 60°C for 30 s, followed by a melt curve (65°C followed by 95°C (5 s at 0.5°C/cycle)). All samples were run in triplicate in 96-well plates (Bio-Rad, Inc.) with a Bio-Rad CFX96 System (Bio-Rad). Single peaks in melting curve analyses were used to confirm gene-specific amplification and rule out non-specific amplification and primer-dimer generation. Relative virus titers were calculated using the average Ct values and the comparative ∆Ct (∆Ct = average endogenous control Ct values – average virus Ct values).

**TABLE 3:** Primers were used to measure relative *Iflavirus* titers using qPCR

Primer	Sequence (5' to 3')	Target virus	Amplicon (bp)	Primer concentration (μM)
BrBVq_RdRp_F	GCTCTGACTGAACTCCAAGC	*Brevicoryne brassicae Iflavirus* (RdRp region)	120	0.8 for both primers
BrBVq_RdRp_R	TCATGCGCATTTTTGGGCC			
IspVq_F	CCCTCCTAGCATGACCGATG	*Illinoia liriodendri Iflavirus* (VP region)	120	0.5 for both primers
IspVq_R	TTAGCTCGAGCAGGCTCTTC			
G3PDH_F	CGGGAATTTCATTGAACGAC	Aphid reference gene	120	0.4 for F primer
G3PDH_R	TCCACAACACGGTTGGAGTA			0.35 for R primer

### *Iflavirus* prevalence and heritability

To assess the prevalence of BrBV, IspV, UeV, and Usp in field populations, we screened single adult field-collected aphids (parental lines, P) using PCR (as above). We then assessed *Iflavirus* maintenance by PCR screening one to three individual adult aphids after one (F_1_) and three (F_3_) generations post-colonization using laboratory-grown plants (Table S6).

For all subsequent transmission experiments, we used three lines of *Iflavirus*-positive aphids showing heritable infections for more than 30 generations in the laboratory [BrBV(+) *B. brassicae* aphids (lines A132 and A134) reared on cabbage (*Brassica oleracea*) and IspV(+) *I. liriodendri* aphids (line A76) reared on shepherd’s purse (*Capsella bursa-pastoris*)]. One *I. liriodendri* IspV(–) line (A65) reared on shepherd’s purse, and one *L. pseudobrassicae* BrBV(–) line reared on cabbage (A130) were used as negative controls. Unfortunately, no *Iflavirus*-negative *B. brassicae* lines were successfully established in the lab.

We assessed heritability as vertical transmission rates using two lines of BrBV(+) *B. brassicae* aphids (A132 and A134) and one line of IspV(+) *I. liriodendri* aphids (A76), totaling four biological replicates per line (reproductive females). We first placed individual 4th instar nymphs in a Petri dish containing a detached leaf of the host plant and allowed them to reach parthenogenic reproduction. At 24 h, the progeny of each female was immediately collected from the posterior ends of their mothers while being born and transferred to new detached leaves. The reproductive females that produced more than five nymphs were stored in Eppendorf tubes at −80°C and then screened for virus status by PCR, as above. The F_1_ nymphs of single reproductive females testing positive for *Iflavirus* infection were maintained on lab-grown plants for 10–14 days until reaching adulthood. Individual F_1_ adults were stored in Eppendorf tubes at −80°C until screened for virus status by PCR, as above. In addition, we used RNA obtained from eight individual *B. brassicae* adults (reproductive females from lines A132 and A134) to measure relative BrBV adult body titers (qPCR). Differences in relative titers (∆Ct) between BrBV(+) lines were analyzed using a two-tailed Mann-Whitney test (*P* < 0.05) using Data GraphPad Prism v.10.0 (https://www.graphpad.com/).

### Within species horizontal transmission of iflaviruses

To investigate whether cohabitation enhances transmission rates of BrBV and IspV, we placed individual 4th instar nymphs (*B. brassicae* and *I. liriodendri*, respectively) in a Petri dish containing a detached leaf of the appropriate host plant and allowed each to reach parthenogenic reproduction. The reproductive females were allowed to cohabitate with their progeny for 4 days before removing all the F_1_ nymphs and placing them on fresh cabbage plants. Reproductive females and adult F_1_ aphids were individually placed in Eppendorf tubes at −80°C and later screened for virus status by PCR, as above. We conducted four biological replicates (reproductive females) per aphid line.

To determine whether iflaviruses were present in body secretions of persistently infected aphids and could serve as a potential source of virus for horizontal transmission, we tested honeydew and cornicle secretions from *B. brassicae* and *I. liriodendri* adults for the presence of the virus. We used forceps to gently press the adults’ abdomens and collected honeydew directly from the aphids’ anus and cornicle droplets using glass capillary needles (volume 0.5 µL). Secretions from several individual adults were pooled until we obtained 10–50 µL. Additionally, we tested plant leaf surfaces contaminated with body secretions, where BrBV(+) and IspV(+) aphid lines had been reared for 10 days. Individual leaves with clusters of 8–12 aphids (nymphs and adults) were clipped; we removed all aphids with forceps and washed the leaves with 500 µL of 1× phosphate-buffered saline (PBS) by pipetting up and down on the surface. We extracted total RNA from body secretions and leaf-wash samples and tested them by qPCR, as described above. Adult aphids (whole-body, *n* = 3), from which honeydew and cornicle droplets were collected, served as a positive control for the qPCR.

To evaluate *Iflavirus’s* ability to penetrate the insect cuticle, virus-free *I. liriondendri* nymphs (line A65) were topically exposed to fresh IspV(+) aphid homogenates. Whole-body aphid homogenates were prepared by shaking 10 adults in 1,000 µL of 1× PBS at 4°C using a Bead Mill Homogenizer (Omni, Kennesaw, GA, USA) for 4 min at 3.1 m/s, followed by centrifugation at 12,000 × *g* for 6 min at 4°C. The clarified supernatant was filtered through a 0.22 µm PDVF syringe filter and used immediately. We placed 20–25 2nd and 3rd-instar nymphs in Petri dishes (3.5 cm diameter) with 500 µL of fresh homogenates for 40 min. Petri dishes were stirred every 10 min to ensure all insects came in contact with the homogenates. Whole-body aphid homogenates from virus-positive aphid lines served as the infectious treatment; virus-negative lines served as negative controls; and PBS was used as the homogenate control. Exposed nymphs were then maintained on shepherd’s purse and allowed each to reach parthenogenic reproduction. The exposed reproductive females and their progeny (F_1_) were collected as adults (whole-body, *n* = 3) and tested by qPCR, as above. Three biological replicates of each feeding event were screened for IspV. *B. brassicae* aphids were excluded due to the lack of colonized BrBV-free lines.

To test whether *Iflavirus* oral ingestion results in active infections, we fed virus-free *I. liriondendri* aphids (line A65) on artificial diets (62) supplemented with IspV(+) aphid homogenates prepared as described above. We delivered the homogenate-supplemented diets (mixed 1:1) to 10–15 2nd- and 3rd-instar nymphs via parafilm sachets (400 µL). Homogenate-supplemented diets from virus-positive aphid lines served as the infectious treatment, while virus-negative lines served as negative controls, and a PBS-supplemented diet was used as the homogenate control. RNA was extracted from a 200 µL sample of the homogenate-supplemented diets to confirm the presence of IspV in the inoculum by PCR. To ensure only aphids that fed on the artificial diet were tested, we added 0.1% blue food coloring (FD&C blue #1; Kroger Co., Cincinnati, OH, USA) to all diets and only collected aphids with blue-colored abdomens. Aph–ids were allowed to feed for 24 h before being transferred to new plants for an additional 7 days. We collected pools of 2–3 surviving aphids immediately after the feeding period (time 0) and again after 2 and 7 days on host plants. All aphids from artificial diet assays were stored in Eppendorf tubes at −80°C. Four biological replicates of each feeding event were screened for virus by PCR, as described above. *B. brassicae* aphids were excluded from all feeding assays due to the absence of colonized BrBV-free lines.

Since other aphid heritable viruses can systemically spread through the plant phloem (20, 29), we tested the potential for *Iflavirus* transmission by feeding on the host plant. We placed the roots of a small shepherd’s purse plant in an Eppendorf tube containing a 1:1 mixture of IspV(+) aphid homogenate and 1× PBS, secured the plant to the Eppendorf tube with parafilm, and placed the setup in a Petri dish. We then placed 2nd and 3rd instar nymphs from the IspV(–) *I. liriodendri* line on top of the leaves. Plants placed only in 1× PBS served as negative controls. To prevent surface contamination during the setup, plant leaves were washed with distilled water and a 5% bleach solution before placing the aphids. Aphids were allowed to feed on the leaves of plants whose roots were submerged in homogenates for 3 days, we then collected 1–3 surviving aphids and transferred the remaining surviving aphids to new unexposed plants for an additional 8 days to allow them to reach adulthood and parthenogenically reproduce on the virus-free plants. We collected all the surviving adult aphids (as individuals) and their progeny (in pools of 2–3 nymphs) produced on virus-free plants. Feeding dishes where all aphids died were excluded from the analysis. Aphids collected from six biological replicates (feeding setup) were placed in Eppendorf tubes at −80°C until screened for IspV by PCR, as above.

### Cross-species transmission of iflaviruses

To determine whether iflaviruses are horizontally transmitted between aphid species from different genera known to cohabitate and feed on the same host plants, we kept three BrBV(+) *B. brassicae* and three BrBV(–) *L. pseudobrassicae* adults in a Petri dish containing a cabbage leaf disc for 48 h. White waxy *B. brassicae* individuals were visually distinguished from green *L. pseudobrassicae*. Reproductive females of both species were collected into Eppendorf tubes at −80°C for subsequent testing for BrBV by PCR, as above. Progeny of the BrBV(–) *L. pseudobrassicae* adults was transferred to virus-free plants, allowed to mature, and tested for BrBV by PCR. Negative PCR results were retested by qPCR to confirm that negative results were not due to the PCR limit of detection. In total, we conducted four biological replicates (feeding setups).

To determine whether iflaviruses are horizontally transmitted across species by oral infection through the host plant, we placed a cabbage leaf stem in an Eppendorf tube containing a 1:1 mixture of 1× PBS and BrBV(+) aphid homogenate (prepared as above). The leaf was secured to the Eppendorf tube with parafilm and washed with distilled water and 5% bleach before placing ten 2nd and 3rd instar nymphs of the BrBV(–) *L. pseudobrassicae* (line A130) on top of the leaves. Leaves placed in 1× PBS served as negative controls. Aphids were allowed to feed on the leaves submerged in homogenates for 3 days, and then we collected 1–3 surviving aphids for testing by PCR and transferred the remaining survivors to new unexposed plants for additional 8 days to allow them to reach adulthood and parthenogenically reproduce on the virus-free plants. Aphids collected from six biological replicates (feeding setups) were placed in Eppendorf tubes at −80°C until screened for BrBV by PCR, and, as above, negative PCR results were confirmed via qPCR. We were unable to use artificial diets supplemented with homogenates from the BrBV(+) lines in *L. pseudobrassicae* aphids since we could not verify the feeding status due to their darker green cuticle color.

Last, to explore the BrBV potential to infect other species when all infection barriers are bypassed, we microinjected approximately 500 nL of undiluted BrBV(+) aphid homogenates in BrBV(–) 4th instar *L. pseudobrassicae* nymphs. Aphid homogenates made with BrBV(–) *L. pseudobrassicae* adults and 1× PBS served as negative controls. After microinjection, aphids were reared on fresh cabbage plants. We collected 10 live aphids 1 h after the injection and at 3 and 7 days post-injection (dpi) in Eppendorf tubes, stored at −80°C, and screened for BrBV infections by qPCR. We also collected the F_1_ offspring produced by injected females as pools of 10 nymphs and tested them as above.

### *Iflavirus* influence on host fitness

To determine the effect of *Iflavirus* infection on life history traits in *I. liriodenri* lines [IspV(−) and IspV(+)], individual 4th instar nymphs were isolated in a Petri dish containing a detached leaf of shepherd’s purse and allowed to reach parthenogenic reproduction. We assessed the effects of viral infection on fecundity as the number of offspring produced per female after four days across six biological replicates (reproductive females) per line. To determine the effects of viral infection on development times, we transferred the 1st instar F_1_ nymphs (pools of 8–10) from single parents to new plants and recorded the number of days needed to reach parthenogenic reproduction (six biological replicates per line). We used a two-tailed Mann-Whitney test (*P* < 0.05) to analyze the differences in life history traits between lines using GraphPad Prism v.10.0. We did not test the effects of *Iflavirus* infection in *B. brassicae* due to the lack of BrBV(–) lines.

The production of winged or wingless offspring as a response to environmental conditions is an important factor in the spread of aphid-associated viruses (63). To measure the effects of viral infection on stress-induced wing induction, we randomly assigned 12-day-old *I. liriodenri* adults reared at low densities for three generations (<10 aphids per plant) to Petri dishes (3.5 cm diameter) either as solitary treatment (one aphid per) or as crowding treatment (12 aphids per dish) for 16 h. Aphids were then placed on a virus-free plant for 3 days and allowed to produce offspring. On day 4, we removed the reproductive adults from the plant and allowed their offspring to reach adulthood. We recorded the percentage of offspring that developed across four biological replicates (treatment per line) and analyzed the differences using a two-tailed Mann-Whitney test (*P* < 0.05) in GraphPad Prism v.10.0. As before, we did not test wing induction responses in *B. brassicae* due to the lack of BrBV(–) lines.

### *Iflavirus* titers in aphid tissues

To evaluate infection patterns of BrBV infection across various tissues, we briefly submerged adult *B. brassicae* aphids (10–14 days after emergence) in 70% ethanol and rinsed twice in 1× PBS before dissecting them under a stereomicroscope using sterile tweezers. Heads (including the salivary glands and brain), digestive tract (including bacteriocytes), developing embryos (including ovarioles), and the remaining carcass were separately collected. We pooled tissues of 20 individuals (maintained on the same plant) in 100 µL of PBS, 7 biological replicates each, and stored at –80°C until processing and testing by qPCR, as above. Differences in relative titers between tissues (∆Ct) were analyzed using a Kruskal-Wallis test followed by multiple comparisons (*P* < 0.05) using Data GraphPad Prism v.10.0.

### Immunohistochemistry

We fixed adult BrBV(+) *B. brassicae* and BrBV(–) *L. pseudobrassicae* (negative controls) in 4% paraformaldehyde (Electron microscopy sciences, Hatfield, PA, USA) and stored at 4°C for approximately 1 week. Aphids were then placed in tissue cassettes (Thermo Fisher Scientific, Inc.), processed through a standard dehydration/infiltration series before embedding in Ameraffin LP paraffin (Cardinal Health, Inc., Dublin, OH, USA), and stored at 4°C. We fixed aphid block sections (5 µM) to charged microscope slides (Colorfrost Plus, Thermo Fisher Scientific, Inc.) by heating at 42°C overnight. Sections were put through a standard deparaffinization and rehydration series and incubated in 1× PBS for 10 min. Antigen retrieval was achieved by submerging sections in citrate-EDTA buffer (10 mM citric acid, 2 mM EDTA, 0.05% Tween20, at pH 6.2) at 88°C for 20 min and cooling down the sections at room temperature before blocking with 6% casein (Sigma-Aldrich, St. Louis, MO, USA). We then incubated the sections with a 1:200 dilution of a polyclonal rabbit anti-BrBV capsid peptide antibody (sequence RGDLEFKFVLNSNKFC) (GenScript, Piscataway, NJ, USA) at room temperature for 1 h, followed by sequential incubation with biotinylated goat anti-rabbit, avidin-biotin alkaline phosphatase (VECTASTAIN ABC-AP Staining kit, Vector Laboratories, Inc., Newark, CA, USA), and Vector Red chromogen substrate (Vector Laboratories), according to manufacturer’s instruction. Between each incubation step, sections were rinsed with PBST (1× PBS, 0.1% Tween20) for 5 min. Tissues were counterstained with Meyer’s hematoxylin (Sigma-Aldrich), followed by 0.1% sodium bicarbonate (Sigma-Aldrich), and covered with CC/Mount (Sigma-Aldrich). Rothschild et al. (64), Forbes (65), and Simonet et al. (27) were used to confirm tissue types.

### Analysis of *Buchnera* density

Lastly, we evaluated whether *Iflavirus* titers affect the titers of the obligate bacterial symbiont, *Buchnera aphidicola*. As mentioned above, we used 1 µL of undiluted cDNA template in a 20 µL qPCR containing 0.5 µM of GroES_F (5′-CTTCGTCCGTTGCATGATCGT-3′) and 0.8 μM of GroES_R (5′-TGCAGCAGAACCCGTAAAGAA-3′), which are specific primers targeting the *B. brassicae Buchnera* gene GroEs (a cofactor essential for cell viability), and we followed the same parameters as mentioned above for *Iflavirus* titer. We used cDNA from eight BrBV(+) adults that showed variable titers in previous experiments and simultaneously performed qPCR for BrBV, *Buchnera,* and the aphid G3PDH (endogenous control). We calculated the relative titers of BrBV and *Buchnera* using the comparative ∆Ct method and analyzed the correlation between the relative titers of both symbionts using a Spearman Rank-Order test in Data GraphPad Prism v.10.0.

## Data Availability

The aphid species barcode sequences are available through NCBI accession PV016919–PV016931. Sequencing raw reads are available through NCBI BioProject PRJNA1216975. Iflavirus genomes have been deposited in NCBI under accessions PV031990, PV023937, PV023938, PV023939, BK070016, BK070017, and BK070018 and will become publicly available once accession formalization resumes.

## SUPPLEMENTAL MATERIAL

Figure S1 aem.01606-25-s0001.pdf Relative abundance of viral protein hits associated with different aphid species.

Figure S2 aem.01606-25-s0002.pdf Evolutionary analysis of aphid iflaviruses using a neighbor-joining tree.

Figure S3 aem.01606-25-s0003.pdf Extended maximum-likelihood analysis of aphid iflaviruses.

Figure S4 aem.01606-25-s0004.tiff Relative *Brevicoryne brassicae virus* titers in *Brevicoryne brassicae* adults.

Figure S5 aem.01606-25-s0005.tiff *Brevicoryne brassicae virus* and *Ilinoia Iflavirus* detected in body secretions.

Figure S6 aem.01606-25-s0006.tiff Effect of *Iflavirus* infection on aphids' fitness in genetically independent *Illinoia liriodenri* lines.

Figure S7 aem.01606-25-s0007.tiff Relative *Brevicoryne brassicae virus* titers in aphid tissues.

Figure S8 aem.01606-25-s0008.tiff qPCR primer efficiencies.

Supplemental tables aem.01606-25-s0009.xlsx Tables S1 to S10.

ASM does not own the copyrights to Supplemental Material that may be linked to, or accessed through, an article. The authors have granted ASM a non-exclusive, world-wide license to publish the Supplemental Material files. Please contact the corresponding author directly for reuse.
