# Supplementary material for: Heritable viral symbionts in the family Iflaviridae are widespread among aphids
Source: Appl Environ Microbiol. 2025 Oct 30;91(11):e01606-25. doi: 10.1128/aem.01606-25 (PMC12628778; doi:10.1128/aem.01606-25)
Supplement: Figure S1 — Relative abundance of viral protein hits associated with different aphid species. [file aem.01606-25-s0001.pdf]

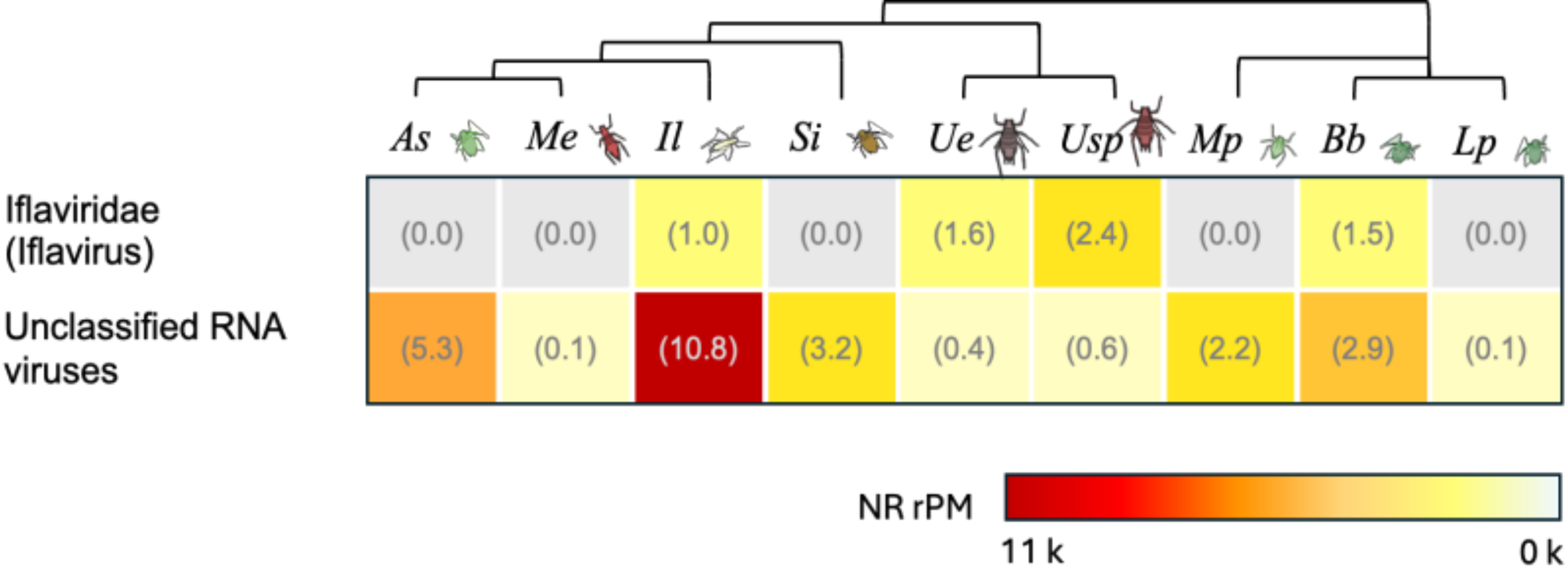

Figure S1 Relative abundance of viral protein hits associated with different aphid species. NR rPM gradient bar indicates the proportion of reads aligning to iflaviruses and unclassified RNA viruses in the NCBI protein database (specific Nr rPM values in parentheses). Aphid species depicted by their phylogenetic association (Aulacorthum solani (As), Macrosiphum euphorbiae (Me), Illinoia liriodendri (Il), Sitobion ibarae (Si), Uroleucon eupatoricolens (Ue), Uroleucon sp. (Usp), Myzus persicae (Mp), Brevicoryne brassicae (Bb), Lipaphis pseudrobrassicae (Lp).
