# Supplementary material for: Heritable viral symbionts in the family Iflaviridae are widespread among aphids
Source: Appl Environ Microbiol. 2025 Oct 30;91(11):e01606-25. doi: 10.1128/aem.01606-25 (PMC12628778; doi:10.1128/aem.01606-25)
Supplement: Figure S2 — Evolutionary analysis of aphid iflaviruses using a neighbor-joining tree. [file aem.01606-25-s0002.pdf]

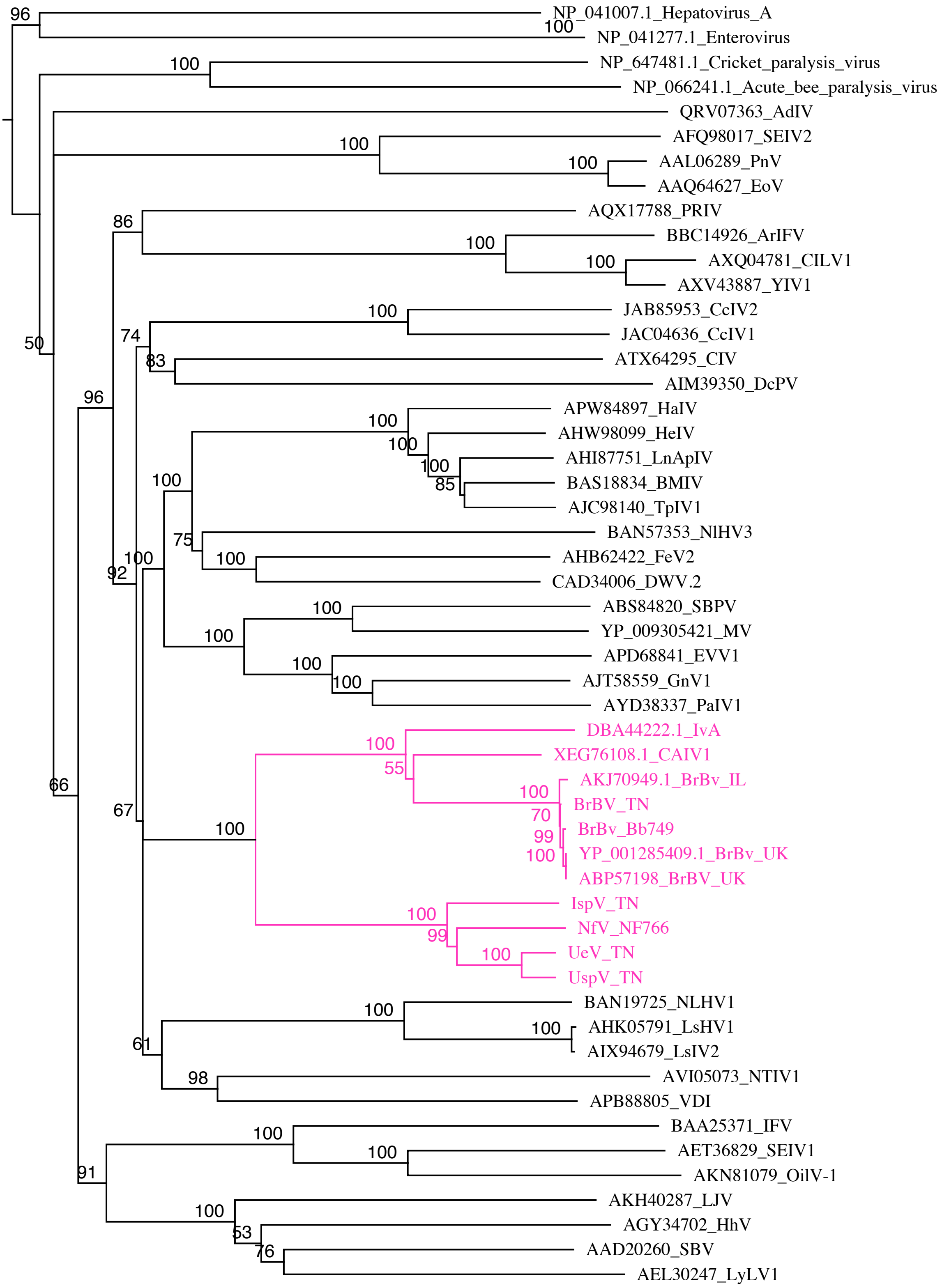

Figure S2 Evolutionary analysis of aphid iflaviruses using a Neighbor-Joining tree. The tree was constructed with the Jukes-Cantor genetic distance model based on an MAFFT alignment of the polyprotein sequences of aphid Iflaviruses (pink clade) and ICTV-recognized members of Iflaviridae. Polyprotein sequences from Dicistroviridae (Acute bee paralysis virus, NP_066241.1, and Cricket paralysis virus, NP_647481.1) and Picornaviridae (Enterovirus C, NP_041277.1, and Hepatovirus A, NP_041007.1) served as outgroups. Bootstrapping values on each node represent the percent from 500 replicates.
