# Supplementary material for: Heritable viral symbionts in the family Iflaviridae are widespread among aphids
Source: Appl Environ Microbiol. 2025 Oct 30;91(11):e01606-25. doi: 10.1128/aem.01606-25 (PMC12628778; doi:10.1128/aem.01606-25)
Supplement: Figure S3 — Extended maximum-likelihood analysis of aphid iflaviruses. [file aem.01606-25-s0003.pdf]

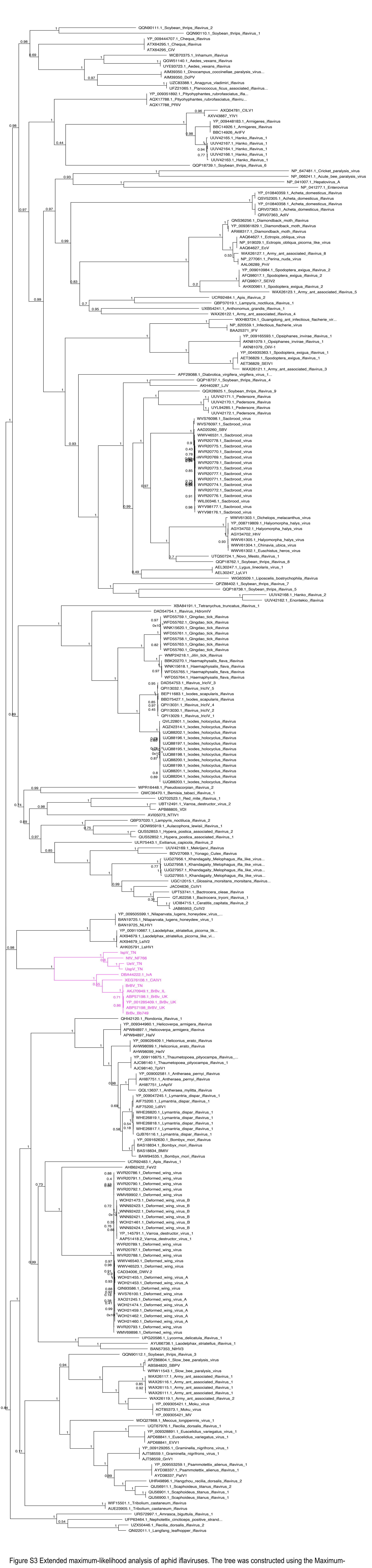

Figure S3 Extended maximum-likelihood analysis of aphid iflaviruses. The tree was constructed using the Maximum-likelihood method with the protein gamma JTT matrix based on an MAFFT alignment of the polyprotein sequences of aphid iflaviruses (pink clade) and sequences from a wide range of iflaviruses infecting arthropods. Polyprotein sequences from Dicistroviridae (Acute bee paralysis virus, NP_066241.1, and Cricket paralysis virus, NP_647481.1) and Picornaviridae (Enterovirus C, NP_041277.1, and Hepatovirus A, NP_041007.1) served as outgroups. Bootstrapping values on each node represent the proportion (0–1) from 500 replicates.
