# Supplementary figures and images for: Heritable viral symbionts in the family Iflaviridae are widespread among aphids
Source: Appl Environ Microbiol. 2025 Oct 30;91(11):e01606-25. doi: 10.1128/aem.01606-25 (PMC12628778; doi:10.1128/aem.01606-25)

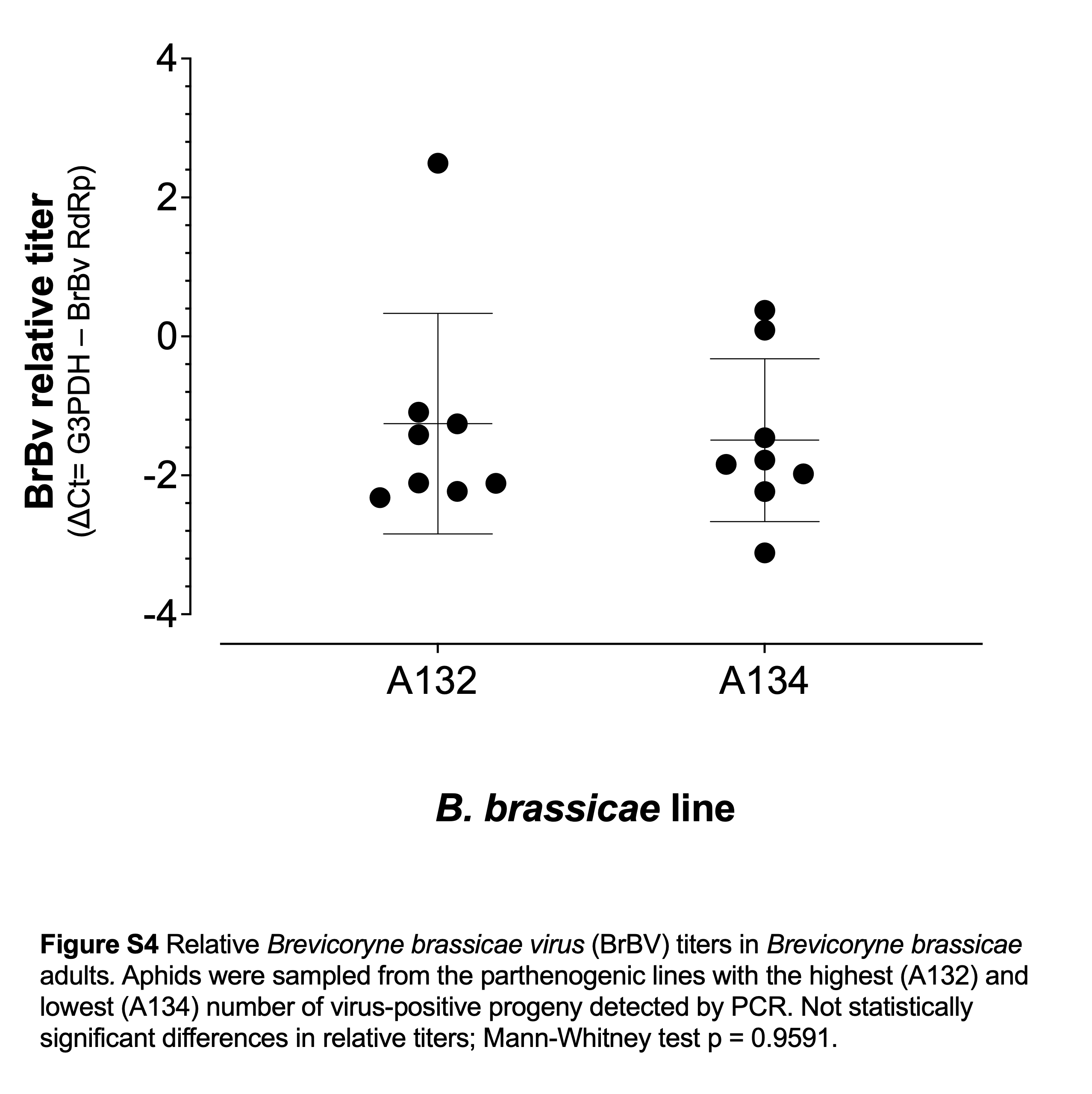

Supplement: Figure S4 — Relative Brevicoryne brassicae virus titers in Brevicoryne brassicae adults. [file aem.01606-25-s0004.tiff]

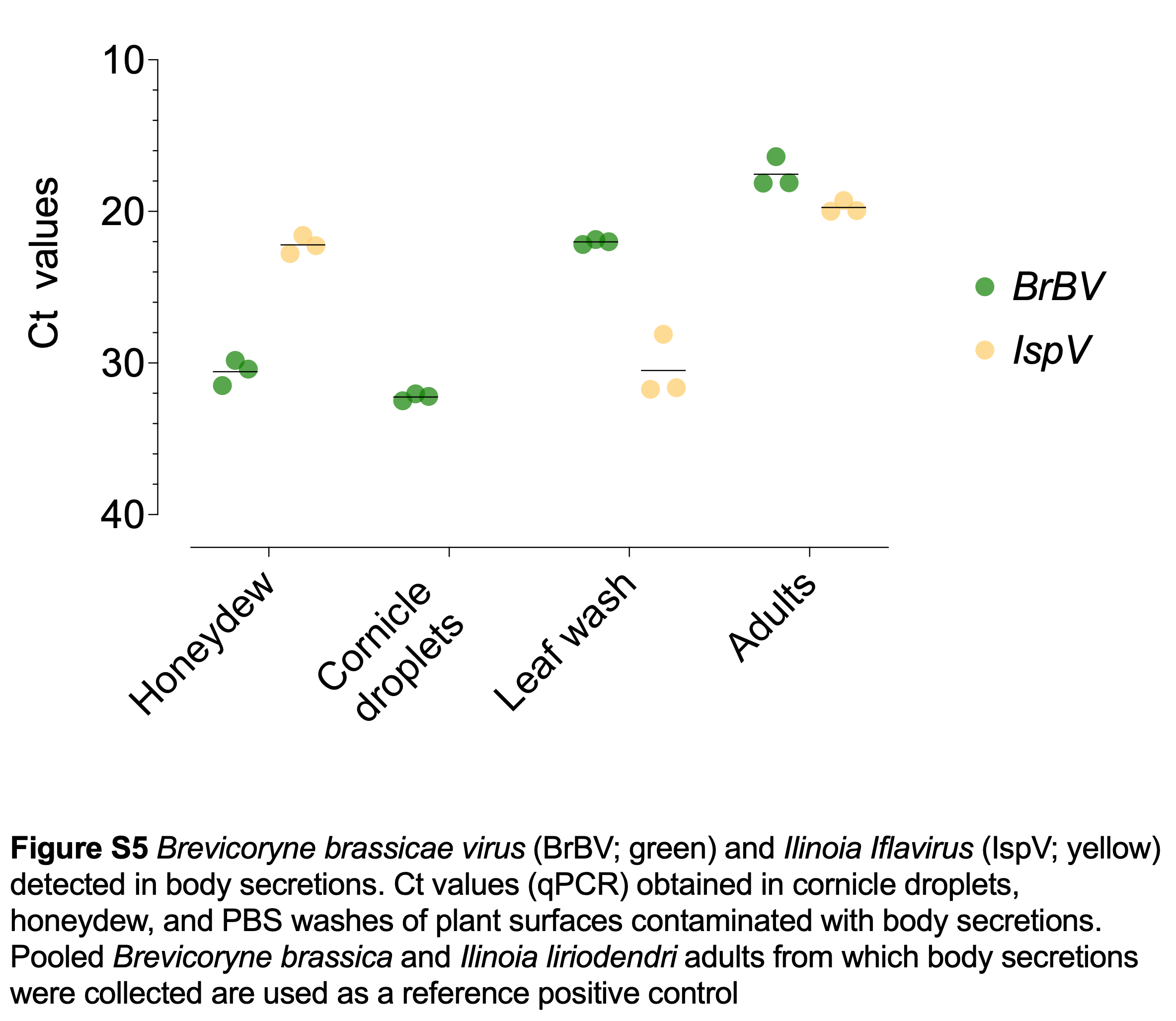

Supplement: Figure S5 — Brevicoryne brassicae virus and Ilinoia Iflavirus detected in body secretions. [file aem.01606-25-s0005.tiff]

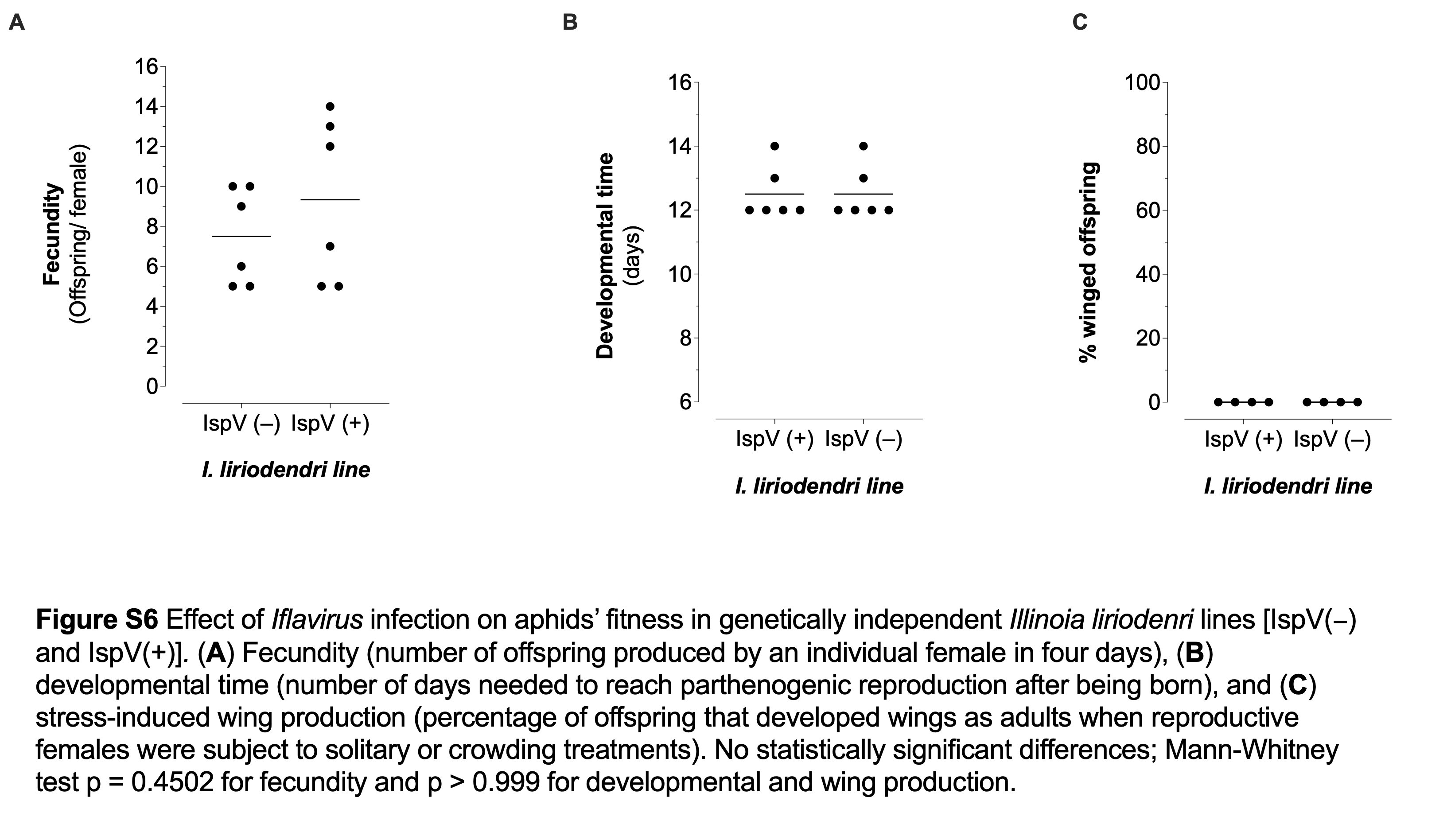

Supplement: Figure S6 — Effect of Iflavirus infection on aphids' fitness in genetically independent Illinoia liriodenri lines. [file aem.01606-25-s0006.tiff]

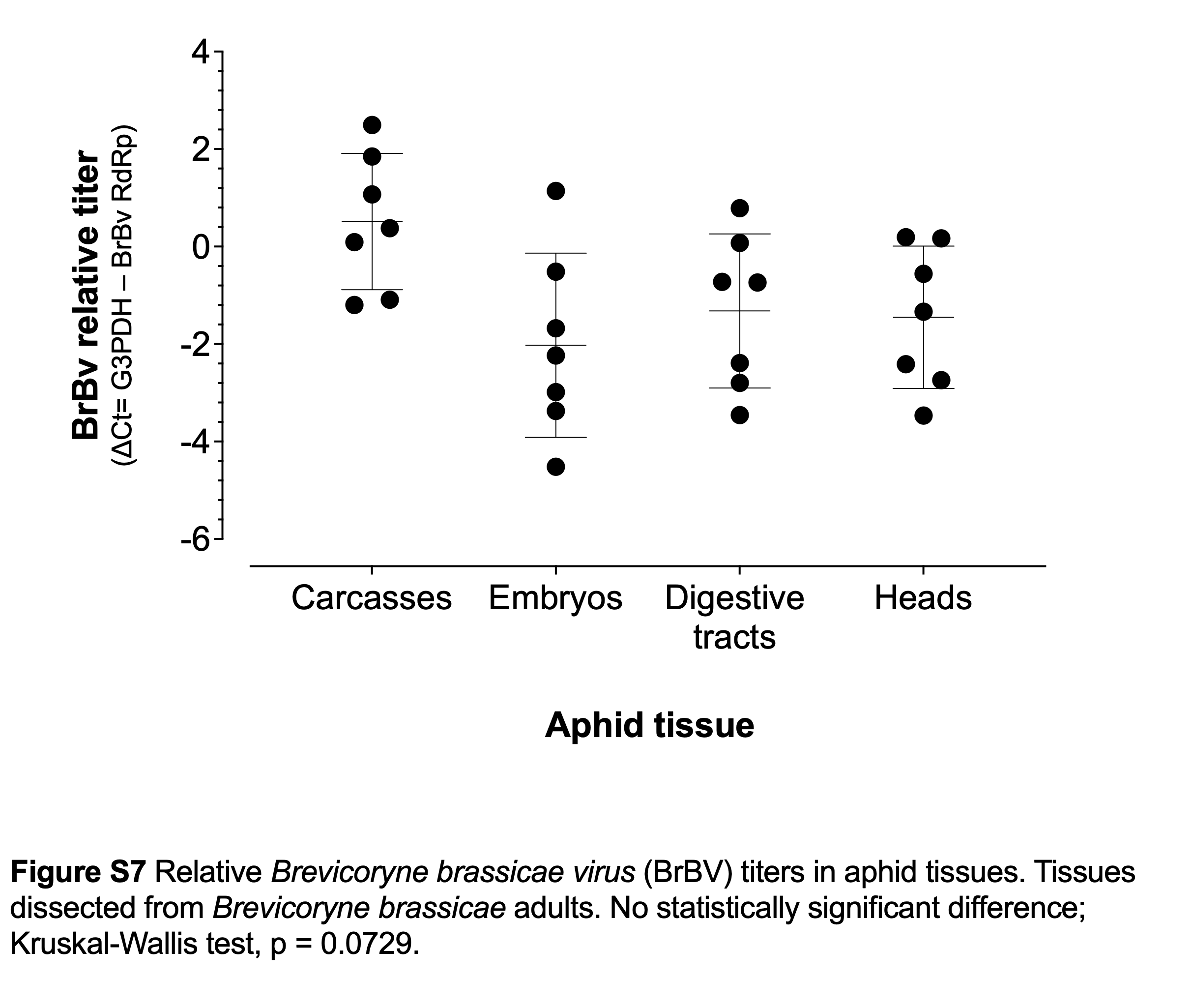

Supplement: Figure S7 — Relative Brevicoryne brassicae virus titers in aphid tissues. [file aem.01606-25-s0007.tiff]

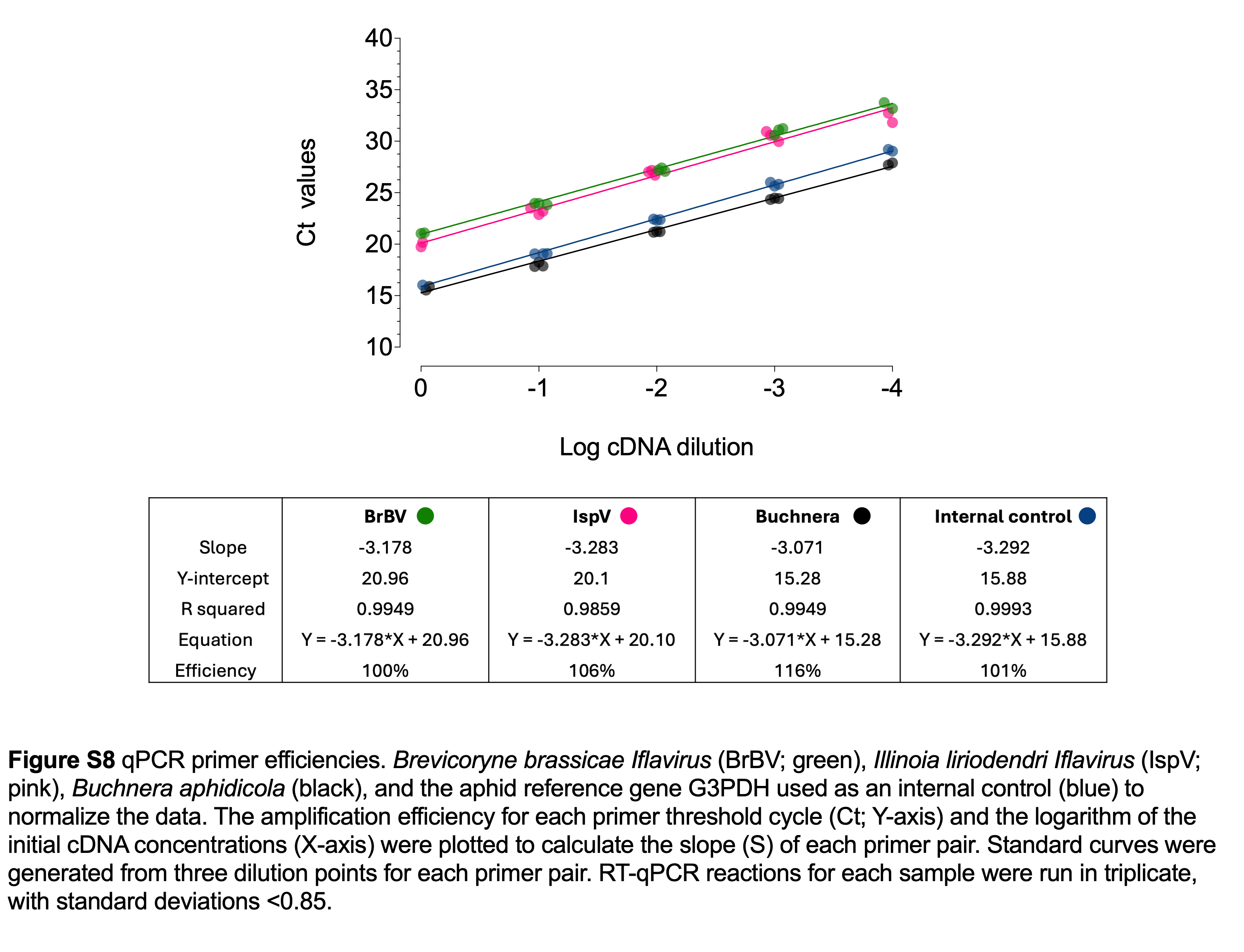

Supplement: Figure S8 — qPCR primer efficiencies. [file aem.01606-25-s0008.tiff]
